# Therapeutic effects of traditional Chinese medicine Hua-Feng-Dan in a rat model of ischemic stroke involve renormalization of gut microbiota

**DOI:** 10.3389/fphar.2025.1485340

**Published:** 2025-01-27

**Authors:** Xiaoxia He, Xiaofeng Yuan, Qilin Shu, Yayang Gao, Youli Chen, Yao Liu, Jian Xu, Yongping Zhang, Guoqiong Cao

**Affiliations:** ^1^ College of Pharmaceutical Sciences, Guizhou University of Traditional Chinese Medicine, Guiyang, China; ^2^ Zunyi Liao Yuan He Tang Pharmaceutical, Zunyi, China; ^3^ National Engineering Technology Research Center for Miao Medicine, Guizhou University of Traditional Chinese Medicine, Guiyang, China; ^4^ Guizhou Engineering Technology Research Center for Processing and Preparation of Traditional Chinese Medicine and Ethnic Medicine, Guizhou University of Traditional Chinese Medicine, Guiyang, China

**Keywords:** Hua-Feng-Dan, middle cerebral artery occlusion, gut microbiome, 16S rRNA sequencing, metabolomics

## Abstract

Hua-Feng-Dan is a traditional Chinese medicine used to treat ischemic stroke, but little is known about its therapeutic mechanism. This study explored whether and how the mechanism involves readjustment of gut microbiota. Rats were subjected to middle cerebral artery occlusion as a model of ischemic stroke or to sham surgery, then treated or not with Hua-Feng-Dan. The different groups of animals were compared in terms of neurological score, cerebral infarct volume, brain edema, brain and gut histopathology to assess stroke severity. They were also compared in terms of indices of intestinal barrier permeability, inflammation and oxidative stress, brain metabolites as well as composition of the gut microbiota and their metabolites. Hua-Feng-Dan significantly reduced cerebral infarct volume and brain water content and improved neurological score, ischemic brain histopathology, and gut histopathology. It partially reversed stroke-induced intestinal barrier disruption and leakage, inflammation, dyslipidemia and oxidative stress, as well as the stroke-induced increase in pathogenic gut microbiota (e.g., *Escherichia-Shigella*, *Enterococcus, Clostridium_innocuum_group*) and decrease in beneficial microbiota (e.g., *Lachnospiraceae*, *unclassified__f__Lachnospiracea* and *Ruminococcus_torques_group*). The treatment altered levels of 39 and 38 metabolites produced during gut microbial and brain tissue metabolism respectively, mainly of amino acids, nucleosides, short-chain fatty acids, and essential fatty acids. Levels of factors related to inflammation and intestinal barrier permeability correlated positively with relative abundance of *Escherichia-Shigella* and *Clostridium_innocuum_group*, and negatively with 4-(glutamylamino) butanoate, 2-hydroxy-3-methylbutyric acid, dihomo-α-linolenic acid, dihomolinoleic acid, and 10-nitrolinoleic acid. Conversely, levels of 4-(glutamylamino) butanoate, 2-hydroxy-3-methylbutyric acid, and 10-nitrolinoleic acid correlated positively with relative abundance of *unclassified__f__Lachnospiracea.* Our results suggest that Hua-Feng-Dan may mitigate ischemic stroke injury by renormalizing gut microbiota and restoring gut barrier function, gut metabolism, thereby helping to alleviate inflammatory, neurological damage, and brain metabolic disorders.

## 1 Introduction

Ischemic stroke (IS), which accounts for 87% of all cases of stroke, involves excitatory amino acid toxicity, oxidative stress, inflammatory responses, and neuronal apoptosis (Paul and Candelario-Jalil, 2021). Most patients experience neurological deficits like hemiparesis, aphasia, and altered level of consciousness, accompanied by gastrointestinal symptoms such as constipation diarrhea (Li et al., 2017). IS can even lead to vascular cognitive impairment and vascular dementia in some patients (Golubnitschaja et al., 2022). Treatments for IS remain limited (Saini et al., 2021), with the two most widely used being thrombolysis with recombinant tissue plasminogen activator and endovascular thrombectomy (Feigin et al., 2021; Lavados et al., 2007). In practice, these therapies benefit a relatively small proportion of patients because they must be administered within a short interval after stroke onset in order to be effective (Emberson et al., 2014; Pinzon and Wijaya, 2020; Wu et al., 2018).

A potential alternative treatment is Hua-Feng-Dan, a traditional Chinese medicine that has been used for 380 years (Liu, 2015) and comprises numerous traditional Chinese medicines, including Tianma (*Gastrodia elata*), Jingjie (*Nepeta cataria*), Cangzhu (*Atractylodes lancea*), Jiangchan (*Bombyx mori)*, Quanxie (*Buthus martensii*), Shexiang (*Moschus berezovskii)*, cinnabar (HgS), and realgar (As_2_S_2_). Hua-Feng-Dan has been used to treat stroke and acute cerebral infarction, in addition to cerebral atrophy, Alzheimer’s disease, Parkinson’s disease and epilepsy (Chen and Gou, 2017; Fan et al., 2022; Lan et al., 2013). The therapeutic mechanism of Hua-Feng-Dan remains unclear, although there have been reports on comparing whether Hua-Feng-Dan with and without realgar and cinnabar rescued loss of dopaminergic neurons, improved behavioral dysfunction and attenuated microglial activation involves reorganization of the gut microbiota (Chen et al., 2020; Hu et al., 2020): the formulation has been shown to increase the relative abundance of *Prevotellaceae* and *Lactobacillaceae* in the gut, its component cinnabar has been shown to increase relative abundance of *Verrucomicrobiaceae* and *Firmicutes* (Liu et al., 2023), and its component *G. elata* is known to reduce cerebral infarction volume and dampen inflammatory responses through mechanisms that involve alterations in gut flora and their metabolism (Ding et al., 2022; Liu et al., 2021).

Here we explored in detail whether Hua-Feng-Dan exerts its therapeutic effects against IS by reversing known stroke-induced changes in the composition of gut microbiota and their metabolism (Ding et al., 2022; Zhang et al., 2023), which have been shown to elevated levels of lipopolysaccharide (LPS) and pro-inflammatory cytokines, contributing to permeabilize the gut and blood-brain barrier (Hu et al., 2022; Padhi et al., 2022; Qian et al., 2023), potentially through processes in which downregulation of tight junction proteins permeabilizes the gut and allows pro-inflammatory cytokines (IL-1β, IL-6, TNF-α) to enter the circulatory system and promote systemic inflammation and neuronal death (Chen R. et al., 2019; Duris et al., 2018; Ma et al., 2017; Sarkar and Banerjee, 2019; Zhao et al., 2021). Contributing to these processes may be metabolites of gut microbiota such as LPS, short-chain fatty acids (SCFAs), bile acids, and *N*-oxidized trimethylamine (Chen R. et al., 2019; Nam, 2019). Therefore, we measured here the effects of Hua-Feng-Dan on stroke injury, the gut microbiome and its metabolism, and brain tissue metabolism in a rat model of IS. We also examined potential correlations between changes in the microbiome and its metabolism with indices of intestinal barrier permeability and inflammation.

## 2 Materials and methods

### 2.1 Hua-Feng-Dan preparation

Hua-Feng-Dan is supplied by Zunyi Liao Yuan He Tang Pharmaceutical (batch 20230601, Zunyi, China). The prescription of Hua-Feng-Dan is as follows: Yaomu, cinnabar, Moschus, Perilae Folium, Bombyx Batryticatus, Scorpio, Arisaematis Rhizoma Preparatum, Atractylodis Rhizoma, realgar, borax, Crotonis Semen Pulveratum, Borneolum Syntheticum, Gastrodile Rhizoma, Schizonepetae Herba, and Santali Albi Lignum (Table 1). In addition to the initial three drugs, the remaining twelve drugs are finely crushed into powder (100 mesh, >95%), mixed with Yaomu and glutinous rice powder (100 mesh, >95%), and formed into pills by adding water; cinnabar (200 mesh, >95%) and Moschus are rapidly ground well and uniformly spread on the surface of the pills to obtain vermilion-red water pills, 0.12 g/pill.

**TABLE 1:** Hua-Feng-Dan prescription compositions.

No.	Traditional Chinese medicine	Pinyin	Type	Latin name or chemical formula	Family	Dosage (g)
1	Gastrodile Rhizoma	Tianma	Herb	*Gastrodia elata* Bl.	Orchidaceae	72.8
2	Arisaemtis Rhizoma Preparatum	Zhitiannanxing	Herb	*Arisaema erubescens* (Wall.) Schott	Araceae	36.7
3	Schizonepetae Herba	Jingjie	Herb	*Nepeta cataria* L.	Lamiaceae	18.3
4	Atractylodis Rhizoma	Cangzhu	Herb	*Atractylodes lancea* (Thunb.) DC.	Asteraceae	36.7
5	Crotonis Semen Pulveratum	Badoushuang	Herb	*Croton tiglium* L.	Euphorbiaceae	12.5
6	Perilae Folium	Zisuye	Herb	*Perilla frutescens* (L.) Britt.	Lamiaceae	162.9
7	Santali Albi Lignum	Tanxiang	Herb	*Santalum album* L.	Santalaceae	3.3
8	Arisaematis Rhizoma	Tiannanxing	Herb	*Arisaema erubescens* (Wall.) Schott	Araceae	28.2
9	Pinelliae Rhizoma	Banxia	Herb	*Pinellia ternate* (Thunb.) Breit.	Araceae	28.2
10	Typhonii Rhizoma	Baifuzi	Herb	*Typhonium giganteum* Engl.	Araceae	28.2
11	Aconiti Radix	Chuanwu	Herb	*Aconitum carmichaelii* Debx.	Ranunculaceae	28.2
12	Curcumae Radix	Yujin	Herb	*Curcuma wenyujin* Y. H. Chen et C. Ling	Zingiberaceae	14.1
13	Moschus	Shexiang	Animal	*Moschus berezovskii* Flerov	Cervidae	2.5
14	Bombyx Batryticatus	Jiangchan	Animal	*Bombyx mori* Linnaeus	Bombycidae	72.8
15	Scorpio	Quanxie	Animal	*Buthus martensii* Karsch	Buthidae	36.7
16	Cow Bile	Niudanzhi	Animal	*Bos taurus* *domesticus* Gmelin	Bovidae	126.7
17	Cinnabar	Zhusha	Mineral	HgS	—	24.2
18	Realgar	Xionghuang	Mineral	AS2S2	—	26.7
19	Borax	Pengsha	Mineral	Na2B4O7·10H2O	—	72.5
20	Borneolum Syntheticum	Bingpian	Mineral	C10H18O	—	36.7
21	Medicated Leaven	Shenqu	—	—	—	0.14

The Yaomu is made by crushing Typhonii Rhizoma, Pinelliae Rhizoma, Arisaematis Rhizoma, Aconiti Radix and Curcumae Radix into a fine powder (100 mesh, >95%), thoroughly mixing them, and then adding into Cow Bile and Medicated Leaven (Shen-Qu) for fermentation. To characterize the compositions of Hua-Feng-Dan, we utilized UPLC-Q-Exactive (Thermo, MA, United States) to detect the water-soluble sample of Hua-Feng-Dan (Supplementary Figure S1; Supplementary Table S1).

### 2.2 Animals

Male Sprague-Dawley rats free of specific pathogens (180–220 g) were purchased from the Institute of Laboratory Animals at the Guizhou University of Traditional Chinese Medicine (Guizhou, China), operating under license SCXK (Qian) 2021-0003. Animal procedures were approved by the Ethics Committee for Animal Experiments at Guizhou University of Traditional Chinese Medicine (approval 20230067). Rats were housed in autoclaved, ventilated cages with autoclaved bedding and sterilized feed. They were maintained on a 12-h light/dark cycle at a temperature of 23°C ± 2°C and relative air humidity of 60%–70%.

### 2.3 Experiment design

The 120 rats were randomly assigned to 20 rats each in sham-operated (Sham), model (MCAO), Hua-Feng-Dan at low dose (HFD-L, 0.162 g/kg/day), Hua-Feng-Dan at middle dose (HFD-M, 0.324 g/kg/day), Hua-Feng-Dan at high dose (HFD-H, 0.648 g/kg/day), and nimodipine (NMDP, 0.19 g/kg/day) groups. Rats were randomized to receive, by oral gavage, saline instead of drug treatment or one of the following drugs dissolved in saline: the calcium channel blocker nimodipine (NMDP, batch 211274, Yabao Pharmaceutical, Shanghai, China), which is widely used to ischemic cerebrovascular disease; and Hua-Feng-Dan. 10 days was the period of the treatment, with modeling conducted 1 h after the drug administration on the 7th day. Three rats were used for 2,3,5-triphenyltetrazolium chloride (TTC) staining, three rats were used for histopathological test, four rats were utilized for brain water content, four rats were utilized for brain metabolomics and 16S rRNA sequencing analysis, six rats were chosen to undergo enzyme-linked immunosorbent assay (ELISA) and gut metabolomics analysis in each group.

### 2.4 Middle cerebral artery occlusion model establishment

Rats were fasted for 12 h, anesthetized with isoflurane inhalant (4% for induction; 1.5% for maintenance), and placed in a supine position. Throughout the following procedures, animals were placed on a thermostatic heating pad to maintain their body temperature at about 37°C. A longitudinal incision was made along the middle of the neck, then the fascia and muscles were separated to expose the left common carotid artery, internal carotid artery, and external carotid artery. The left common and external carotid arteries were ligated, while the internal carotid was clamped with a microvascular clamp. A nylon wire of diameter 0.25 mm was inserted from the left common carotid into the internal carotid for approximately 18.5 ± 0.5 mm, then the incision was rapidly closed. After 2 h of occlusion, the nylon wire was pulled out a certain length to allow reperfusion. Rats were kept under the oven lamp until they recovered from anesthesia. As controls, sham-operated rats underwent the same procedures except that no nylon wire was inserted.

### 2.5 Neurological score

Rats were assessed for nerve damage using a previously described scoring system (Longa et al., 1989). Points were assigned as follows: 0, normal walking without apparent nerve damage; 1, weak contralateral forelimb and inability to fully extend it; 2, circling toward the contralateral side; 3, tilting toward the contralateral side; and 4, failure to walk spontaneously and diminished or no consciousness. Only animals scoring 1–3 immediately after awakening from reperfusion were randomized to receive drug treatments. The assessment was repeated at 72 h after reperfusion.

### 2.6 Cerebral infarct volume

At 72 h following reperfusion, the brains were frozen at −20°C for 20 min and then sliced into six sections along the coronal plane, immersed in 2% (v/v) TTC (batch 23206277, Sigma, St Louis, MO, United States), stained at 37°C without light for 15 min to visualize the infarct volume, and fixed in 4% paraformaldehyde solution (batch 22182401, Biosharp, Anhui, China). Infarct volume and total brain volume across all six sections were determined using ImageJ 2.9.0 (US National Institutes of Health, Bethesda, MD, United States), and extent of cerebral infarction was calculated as.

$$\text{Cerebral infarction extent }\left(\%\right)=\left(\text{infarct volume }/\text{ whole brain volume}\right)\times 100\%.$$

### 2.7 Assessment of the brain water content

The brains of rats were immediately removed 72 h after modeling. Carefully removing the leptomeninges, cerebellum, and brain stem, any liquid that remained on the surface was absorbed using filter paper. The cerebral hemispheres of the infarcted side were promptly weighed to determine their wet weight using an analytical balance. The brain was placed in an electric thermostat where it was baked at 110°C for 24 h; this dry weight was recorded. The water content of ischemic brain was calculated using the following formula:

$$\text{Brain water content }\left(\%\right)=\left(\text{wet weight}-\text{dry weight}\right)\;/\text{ wet weight}\times 100\%.$$

### 2.8 Hematoxylin-eosin and terminal deoxynucleotidyl transferase dUTP nick end labeling staining

Brain and colon tissues were fixed in 4% paraformaldehyde solution, embedded in paraffin, cut into sections 5 μm and 4 μm thick, respectively, and stained with hematoxylin-eosin (H&E, batch G1003, Servicebio, Hubei, China). Additionally, we stained brain sections utilizing the TUNEL assay kit (batch G1504, Servicebio). Histopathology in the cerebral cortex, hippocampus and colon was assessed using an orthoptic light microscope (Eclipse E100, Nikon, Tokyo, Japan) equipped with a DS-U3 imaging system (Nikon). Observations and image acquisition of TUNEL were performed using an ECLIPSE C1 orthogonal fluorescence microscope (Nikon). The number of TUNEL positive cells at three distinct locations within each section was quantified using Image-Pro Plus 6.0 (Media Cybernetics, MD, United States).

### 2.9 Systemic inflammation, gut barrier permeability, total cholesterol and oxidative stress

At 72 h of reperfusion, rat serum was assayed for tumor necrosis factor (TNF)-α, interleukin (IL)-1β and IL-6 as indices of inflammation. It was also assayed for lipopolysaccharide (LPS), diamine oxidase (DAO), and d-lactate (d-LA) as indices of permeability of the intestinal epithelium; for total cholesterol (T-CHO) as an index of dyslipidemia; and for total superoxide dismutase (T-SOD) and malondialdehyde (MDA) as indices of oxidative stress. The first six assays were ELISA with kits purchased from Shenzhen Ziker Biological Technology (Shenzhen, China), while the last three assays were utilized kits provided by the Nanjing Jiancheng Bioengineering Institute (Nanjing, China).

### 2.10 Composition of gut microbiomes

After 72 h of modeling, cecal contents were collected into sterile 1.5-mL microcentrifuge tubes, labeled, snap-frozen in dry ice, and stored at −80°C. DNA was extracted from thawed samples using the Fast Pure Stool DNA Isolation Kit (MJYH, Shanghai, China), the variable region V3-V4 within 16S rRNA was amplified using primer 338F-806R through PCR, and the PCR products were used to create a paired-end (PE) library, which was sequenced on a NextSeq 2000 system (Illumina, California, United States). The detailed parameters of the 16S rRNA sequencing can be found in the Supplementary Material. Raw sequences were deposited into the Sequence Read Archive at the National Center for Biotechnology Information under accession code PRJNA1129823 (www.ncbi.nlm.nih.gov).

Sequencing PE reads were split, and double-ended raw sequences were checked for quality using FASTP 0.19.6 software (Chen et al., 2018), then spliced using FLASH 1.2.11 software (Magoč and Salzberg, 2011) and subjected to noise reduction using the plugin DADA2 (Callahan et al., 2016) within the Qiime2 pipeline (Bolyen et al., 2019), resulting in amplicon sequence variants (ASV). The number of sequences from each sample was rarefied to 28,770, and the ASVs were analyzed for species taxonomy based on the Sliva 16S rRNA database (version 138; www.arb-silva.de), utilized naïve Bayes classifier in the Qiime2.

Based on relative abundances of microbial taxa, we calculated α-diversity using MOTHUR 1.30.2 software (Schloss et al., 2009), and inter-group differences were assessed for significance using the Wilcoxon rank sum test. We also calculated β-diversity using principal coordinate analysis (PCoA) based on Bray-Curtis dissimilarities. Bar charts were used to compare gut microbiomes among groups at the levels of phylum, family and genus. “Linear discriminant analysis (LDA) effect size (LEfSe)” (Segata et al., 2011) was used to determine gut microbial taxa with significant differences in abundance from phylum to genus level; differential bacteria were defined as those whose relative abundance differed between groups with a LDA score >4 and *P* < 0.05. Based on the reconstructed gut microbiomes, we predicted functional information of the microbial communities in the samples using PICRUSt2 2.2.0-b software (Douglas et al., 2020). Differences between groups were assessed for significance using the Wilcoxon rank-sum test.

### 2.11 Gut and brain metabolomics analysis

Aliquots of cecal contents (see Section 2.9) and brain tissues weighing 50 ± 5 mg were respectively placed into 2-mL microcentrifuge tubes, to which were added a grinding bead with diameter of 6 mm and 400 µL of methanol/water (4/1, v/v) containing the following internal standard l-2-chlorophenylalanine (0.02 mg/mL, Adamas-beta), respectively. The sample was ground for 6 min using a freezing tissue grinder (-10°C, 50 Hz), cryo-sonicated for 30 min at 5°C (40 kHz), left at -20°C for 30 min, then centrifuged for 15 min at 13,000 *g* at 4°C. Reproducibility of analysis results was assessed by mixing 20 µL equal aliquots of different samples to obtain quality control (QC) samples.

The resulting supernatant was injected onto a Vanquish Horizon ultra-high performance liquid chromatography (Thermo, MA, United States) equipped with a ACQUITY UPLC HSS T3 column (100 mm long, 2.1 mm inner diameter, 1.8 µm pore size; Waters, Milford, MA, United States). The following chromatographic parameters were used: the mobile phase A was 95% water and 5% acetonitrile (containing 0.1% formic acid), while mobile phase B was 47.5% acetonitrile, 47.5% isopropanol, and 5% water (containing 0.1% formic acid), injection volume, 3 μL; column temperature, 40°C. The detailed parameters of the LC-MS program can be found in the Supplementary Material.

Eluted compounds were identified using Q-Exactive HF-X mass spectrometry (Thermo) at a sheath gas flow rate, 50 Arb; auxiliary gas flow rate, 13 Arb; capillary temperature, 325°C; a mass scan range, 70-1,050; full resolution, 60,000; MS/MS resolution, 7,500; collision energy, 20/40/60 Ev; and spray voltage, ±3.5 kV. Raw chromatographic data were corrected for retention time and peaks were aligned using Progenesis QI 3.0 software (Waters). Peaks were identified through comparison with the Human Metabolome Database (HMDB, www.hmdb.ca), Metlin database (https://metlin.scripps.edu), and a self-built database (Majorbio Bio-Pharm Technology CO., LTD., Shanghai, China), based on a mass error threshold of 10 ppm and secondary mass spectrometry match scores. Peak annotations were verified manually. Data were analyzed in both positive and negative ion modes.

The stability of the internal standards was assessed by checking whether the z-score values fell within two times the standard deviation. Principal component analysis (PCA) and orthogonal partial least-squares discriminant analysis (OPLS-DA) were utilized to assess the quality of the LC-MS data. Statistical significance between groups of metabolites was assessed using a two-tailed unpaired Student’s t-test. Differential metabolites screening criteria included OPLS-DA Variable Importance in the Projection (VIP) > 1 and *P* < 0.05. Metabolic pathway annotation was conducted using the KEGG database (www.kegg.jp/kegg/pathway.html). Pathway enrichment analysis was performed using the Python package, and relevant metabolic pathways were identified through Fisher’s exact test.

### 2.12 Statistical analysis

Data were analyzed statistically using SPSS 26.0 software (IBM, Chicago, IL, United States). Data were reported as mean ± standard deviation (SD). Differences between two groups were assessed for significance using Student’s two-tailed *t*-test, while differences among at least three groups were assessed using a one-way ANOVA and Tukey’s *post hoc* tests. Differences were considered statistically significant at *P* < 0.05. Potential correlations between relative abundance of microbiota and levels of gut metabolites were explored using the Spearman algorithm. Data were plotted using GraphPad Prism 9.0 (GraphPad Software, San Diego, CA, United States).

## 3 Results

### 3.1 Hua-Feng-Dan ameliorates neurological deficits and histopathological injuries after ischemic stroke in rats

**FIGURE 1:**
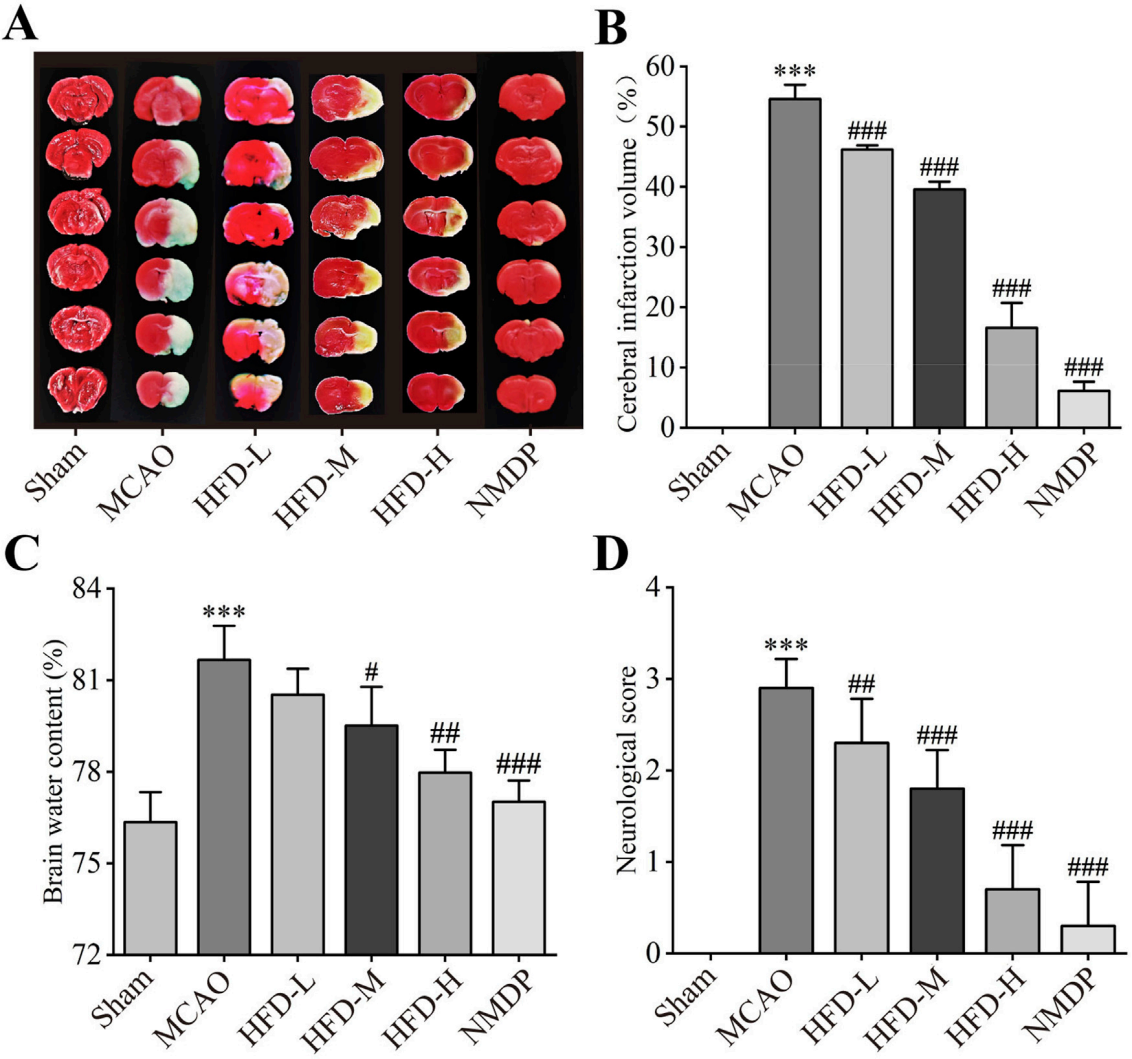
Hua-Feng-Dan ameliorates the size of the brain infarct and the neurological impairments. Animals were subjected to middle cerebral artery occlusion or sham surgery, then left untreated (MCAO, Sham) or treated with nimodipine (NMDP) or Hua-Feng-Dan at a low dose (HFD-L, 0.162 g/kg), intermediate dose (HFD-M, 0.324 g/kg) or high dose (HFD-H, 0.648 g/kg). **(A)** Representative photographs of ischemic halves of brains after TTC staining. **(B)** Extent of cerebral infarction (n = 3). **(C)** Brain water content (n = 4). **(D)** Neurological score (n = 20). Quantitative data are mean ± SD. ***P < 0.001 vs. Sham group; ^##^ *P* < 0.01, ^###^ *P* < 0.001 vs. MCAO group; based on one-way ANOVA and Tukey’s multiple-comparisons *post hoc* test.

As expected, MCAO created a cerebral infarct area on the infarct side of the brain (Figure 1A), which Hua-Feng-Dan significantly reduced in a dose-dependent manner (Figure 1B). In fact, the infarct volume after treatment with the highest dose was comparable to that after treatment with nimodipine. Similarly, the highest doses of Hua-Feng-Dan and nimodipine significantly reduced brain water content following ischemia, while HFD-L group did not differ considerably from MCAO groups (Figure 1C). In a dose-dependent manner, Hua-Feng-Dan also reduced neurological score (Figure 1D).

**FIGURE 2:**
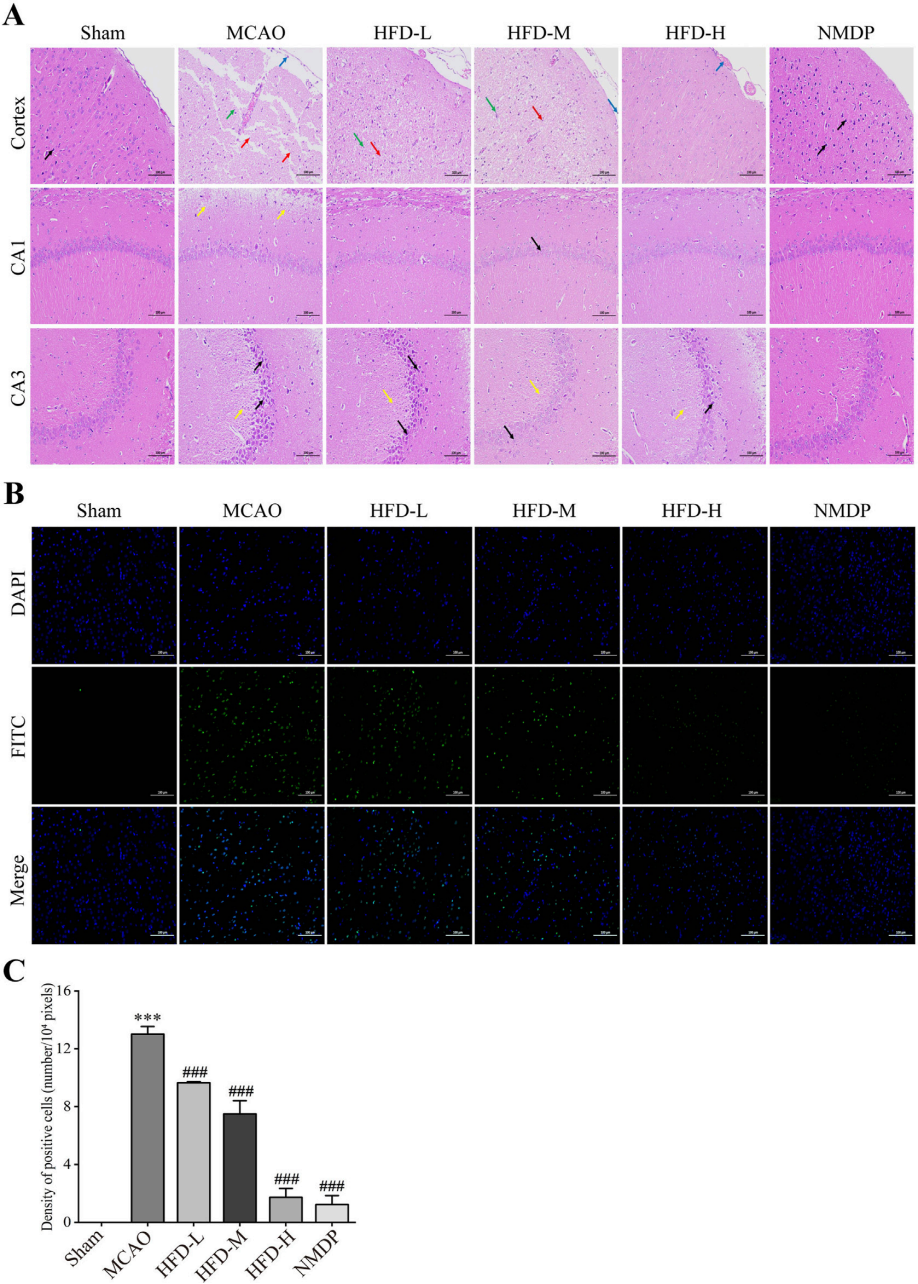
Hua-Feng-Dan alleviates histopathology injury and apoptosis of nerve cells after ischemic stroke in rats. Animals were subjected to middle cerebral artery occlusion or sham surgery, then left untreated (MCAO, Sham) or treated with nimodipine (NMDP) or Hua-Feng-Dan at a low dose (HFD-L, 0.162 g/kg), intermediate dose (HFD-M, 0.324 g/kg) or high dose (HFD-H, 0.648 g/kg). **(A)** Thin sections of brain tissue after hematoxylin-eosin staining (n = 3). Blue arrows represent macrophage infiltration or vascular stasis; red arrows, necrotic neurons or pyramidal cells; green arrows, glial cell proliferation; yellow arrows, vacuoles or septa; and black arrows, crumpled neurons or pyramidal cells. Scale bar, 100 μm. **(B)** Thin sections of brain tissue after terminal dexynucleotidyl transferase (TdT)-mediated dUTP nick end labeling (TUNEL) staining (n = 3). Scale bar, 100 μm. **(C)** TUNEL positive cells density (n = 3).

Hua-Feng-Dan ameliorated the ischemia-induced sparse structuring, neuronal necrosis, tissue disorganization, and invasion of macrophages and glia in the cerebral cortex; and it ameliorated the crumpling, intracellular vacuolization and excessive intercellular spacing of pyramidal cells in the hippocampus (Figure 2A). The mechanism behind nerve cell death was examined by TUNEL staining (Figure 2B). The MCAO group had a significant number of apoptotic nerve cells, and that the damage was decreased in a dose-dependent manner by Hua-Feng-Dan treatment (Figure 2C). These findings presented Hua-Feng-Dan can lessen both the neurological and histopathological damage caused by ischemia.

### 3.2 Hua-Feng-Dan renormalizes the gut microbiome after ischemic stroke in rats

**FIGURE 3:**
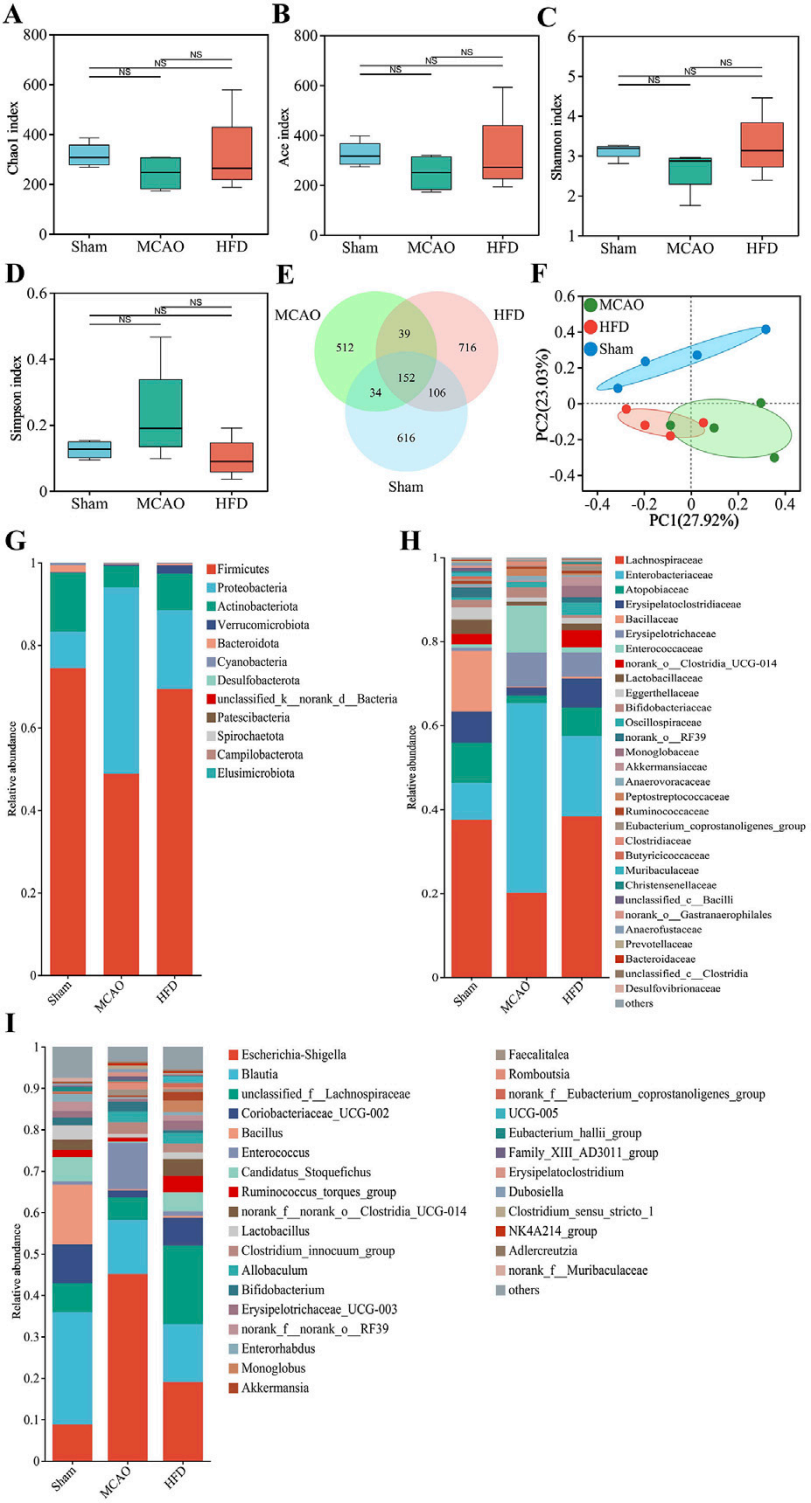
Hua-Feng-Dan renormalizes the gut microbiome after ischemic stroke in rats. Animals were subjected to middle cerebral artery occlusion or sham surgery, then left untreated (MCAO, Sham) or treated with a high dose of Hua-Feng-Dan (HFD, 0.648 g/kg). **(A–D)** Comparison of α-diversity based on the **(A)** Chao1 index and **(B)** Ace index to evaluate the richness of gut flora, and **(C)** Shannon index and **(D)** Simpson index to evaluate the diversity of gut flora. **(E)** Venn diagram of numbers of amplicon sequence variants (ASV) in the different groups. **(F)** Principal coordinate analysis (PCoA) of β-diversity of gut flora based on the Bray-Curtis distance algorithm. **(G–I)** Histograms of relative abundance of gut flora at the level of **(G)** phylum, **(H)** family or **(I)** genus. Data are mean ± SD. Differences were assessed for significance using one-way ANOVA and Tukey’s multiple-comparisons *post hoc* test.

Neither MCAO nor treatment with the highest dose of Hua-Feng-Dan significantly altered the richness or diversity of the gut microbiome (Figures 3A–D), based on analysis of 908 ASVs (616 unique), 737 ASVs (512 unique), and 1,013 ASVs (716 unique) were identified in the Sham, MCAO, and HFD groups, respectively (Figure 3E). Nevertheless, principal coordinate analysis (PCoA) indicated that the MCAO significantly altered the β-diversity of gut microflora, with the highest dose of Hua-Feng-Dan shifting the structure of gut microbiota closer to that of sham-operated animals (Figure 3F). These results suggest that IS can cause dysbiosis of the gut flora, which Hua-Feng-Dan can renormalize.

Specifically, MCAO was associated with a significant increase in relative abundance of *Proteobacteria*, *Enterobacteriaceae*, *Enterococcaceae*, *Erysipelotrichaceae*, *Escherichia-Shigella*, *Enterococcus* and *Clostridium_innocuum_group* (Figures 3G–I). It was also associated with a significant decrease in relative abundance of *Firmicutes*, *Actinobacteriota*, *Lachnospiraceae*, *Atopobiaceae*, *Blautia*, *Bacillus*, *Candidatus_Stoquefichus* and *Lactobacillus.* Hua-Feng-Dan significant reversed these changes in relative abundance, while also increasing the abundance of *unclassified__f__Lachnospiraceae*, *Ruminococcus_torques_group*, *norank__f__norank__o___Clostridia_UCG-014*, *Monoglobus* and *Akkermansia*.

**FIGURE 4:**
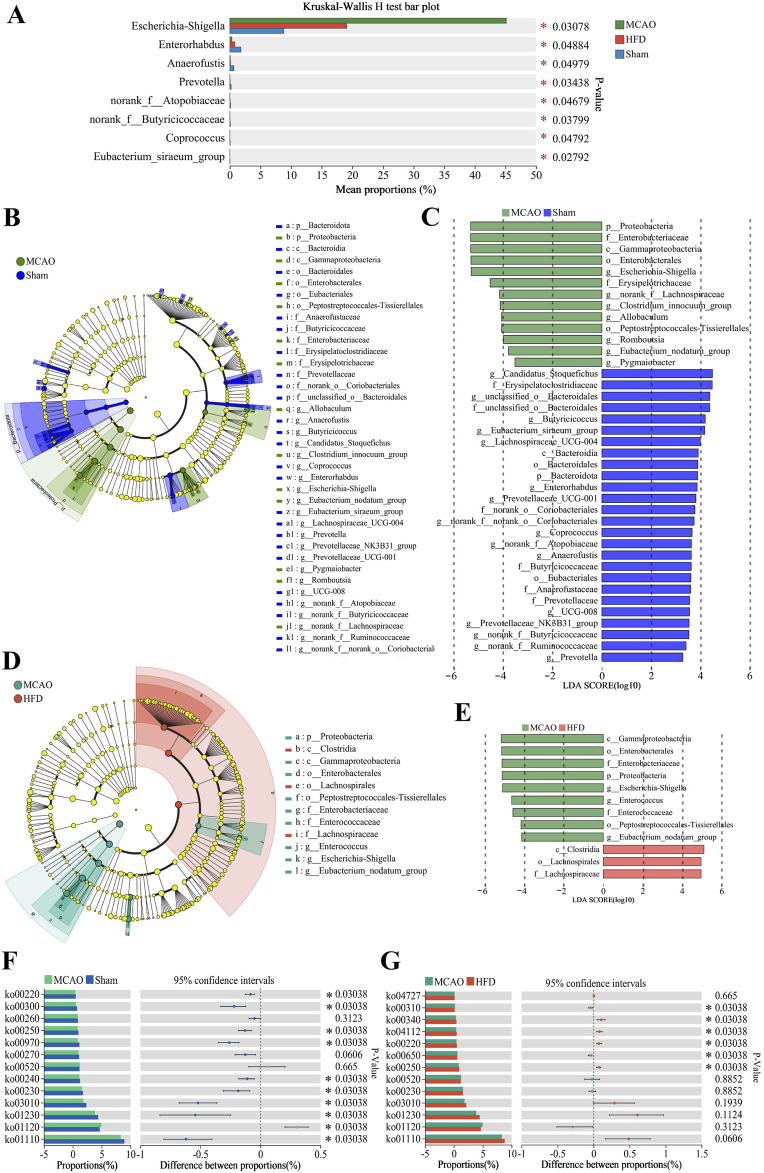
Differences in the composition and metabolism of the gut microbiota due to ischemia injury and treatment with Hua-Feng-Dan. Animals were subjected to middle cerebral artery occlusion or sham surgery, then left untreated (MCAO, Sham) or treated with a high dose of Hua-Feng-Dan (HFD, 0.648 g/kg). Gut microbiomes and their predicted metabolic pathways were compared between Sham and MCAO animals, as well as between MCAO and HFD animals. **(A)** Significance testing of differences in relative abundance of gut microbiota at genus level. **(B–E)** Linear discriminant analysis effect size (LEfSe) analysis to identify microbial species differing significantly in relative abundance in the gut. **(F, G)** Metabolic pathways predicted by phylogenetic investigation of communities by reconstruction of unobserved states (PICRUSt) analysis in the gut microbiota of different groups. Differences were assessed for significance using the Wilcoxon rank-sum test. Data are mean ± SD. *P < 0.05.

Comparison across treated, untreated and sham-operated animals identified several genera whose relative abundance differed the most, among them *Escherichia-Shigella*, *Enterorhabdus*, *Prevotella*, *norank_f__Butyricicoccaceae*, *Coprococcus* (Figure 4A). We identified several species that predominated in untreated animals but not in the other two groups: *p_Proteobacteria*, *f_Enterobacteriaceae*, *g_Escherichia-Shigella*, *g__Clostridium_innocuum_group*, *g__Allobaculum*, *g__Romboutsia* and *g__Enterococcus.* We also identified several species that predominated in treated animals but not in untreated ones, among them *c_Clostridia, o_Lachnospirales* and *f_Lachnospiraceae* (Figures 4B–E).

### 3.3 Hua-Feng-Dan renormalizes the gut microbial metabolism after ischemic stroke in rats

The abovementioned changes in the composition of gut microbiota were associated with predicted changes in microbial function. MCAO was associated with biosynthesis of aminoacyl-tRNAs and amino acids, as well as metabolism involving purines, pyrimidines, alanine, aspartate and glutamate (Figure 4F). Hua-Feng-Dan treatment was also associated with metabolism of alanine, aspartate and glutamate, as well as metabolism of butanoate and histidine, biosynthesis of arginine, and degradation of lysine (Figure 4G).

**FIGURE 5:**
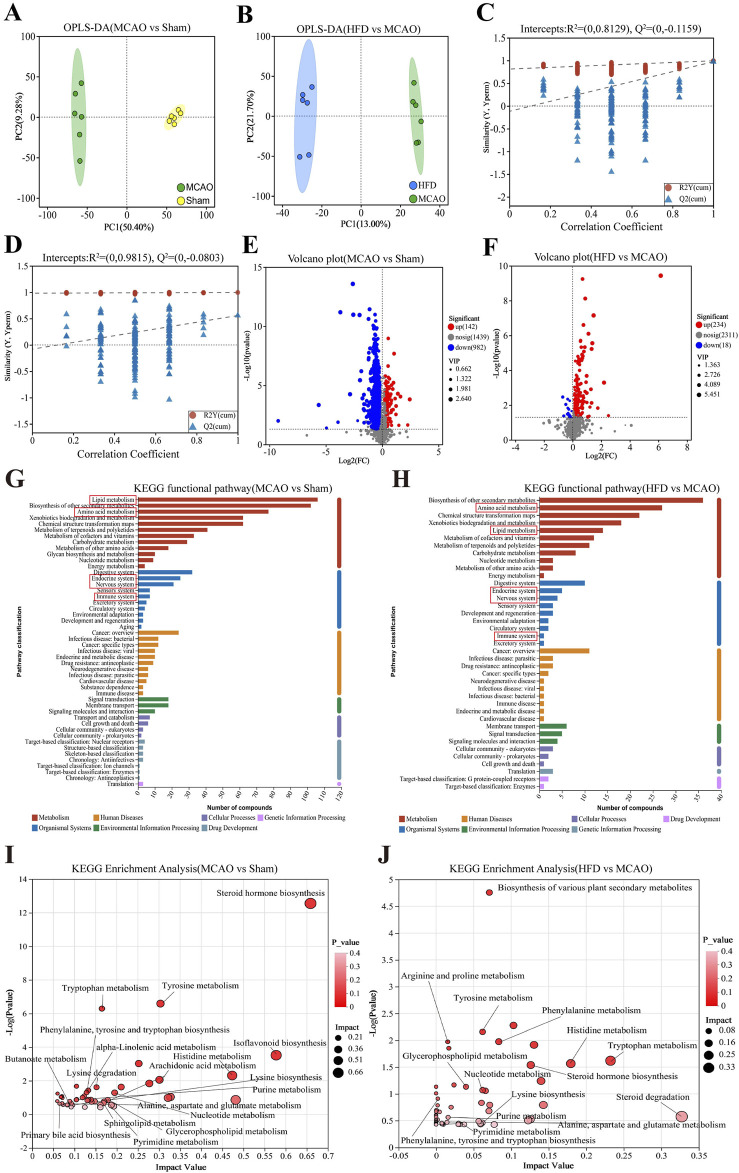
Hua-Feng-Dan renormalizes gut microbial metabolism after ischemic stroke in rats. Animals were subjected to middle cerebral artery occlusion or sham surgery, then left untreated (MCAO, Sham) or treated with a high dose of Hua-Feng-Dan (HFD, 0.648 g/kg). Levels of metabolites were compared between Sham and MCAO animals, as well as between MCAO and HFD animals. **(A, B)** Orthogonal partial least-squares-discriminant analysis (OPLS-DA) in negative ionization mode. **(C, D)** OPLS-DA permutation testing with 200 permutations in negative ionization mode. *R* ^2^ measures goodness of fit, while Q^2^ measures predictive power of the model. **(E, F)** Volcano plots showing gut metabolites whose levels differed significantly in each pairwise comparison (negative ionization mode). **(G–J)** Analysis of Kyoto Encyclopedia of Genes and Genomes (KEGG) pathways involving gut metabolites whose levels differed significantly in each pairwise comparison. **(G, H)** KEGG functional pathways involving the gut metabolites showing significant differences in each comparison. **(I, J)** Bubble plots of differential metabolic pathways involving the differential functional pathways in this figure **(G, H)**. Data are mean ± SD.

The z-score values of the QC samples were within ±2 SD, and the total ion chromatogram peak shapes were well-defined and consistent distributions, suggesting that the equipment is stable and the data quality is reliable (Supplementary Figures S2, S3). To explore these metabolic changes in detail, we performed metabolomics on cecal contents from the different animal groups. Metabolomic profiles differed significantly across untreated, treate d and sham-operated animals (Figures 5A, B), with the data suggesting that Hua-Feng-Dan partially reversed ischemia-induced changes to renormalize gut microbial metabolism. These results are unlikely to reflect overfitting based on permutation testing (Figures 5C, D) and are therefore likely to be reliable. Indeed, volcano plots highlighted obvious differences in metabolite levels across the three groups (Figures 5E, F). Metabolic data in the positive ion mode are presented in the Supplementary Figure S4.

Extrapolating from levels of individual metabolites to entire metabolic pathways, we found that ischemic brain injury significantly affected pathways involving metabolism of lipids and amino acids, biosynthesis of other secondary metabolites, which primarily affect the body’s endocrine, nervous, and immune systems (Figures 5G, H). The MCAO also affected the biosynthesis of steroid hormones, isoflavone, and primary bile acids; metabolism of glycerophospholipids, sphingolipids, arachidonic acid, tryptophan, histidine, nucleotides, α-linolenic acid, and butanoate; and lysine degradation (Figures 5I, J). Hua-Feng-Dan significantly reversed these ischemia-induced changes, while also altering pathways involving degradation of steroids, biosynthesis of plant-derived secondary metabolites, and metabolism of arginine and proline.

Using carefully defined cut-offs (see *Methods*), we identified 39 metabolites whose levels in the gut were significantly altered by brain ischemia and were partially renormalized by Hua- Feng -Dan (Table 2). These potential biomarkers are 3-methylindole and other metabolites known to be involved in metabolic pathways involving amino acids (e.g., prolyl-glutamate, *N*2-succinyl-l-ornithine, *N*-arachidonoyl phenylalanine), SCFAs [e.g., 3-pyridylacetic acid, l-2-amino-3-(1-pyrazolyl)propanoic acid, 2-hydroxy-3-methylbutyric acid, 4-(glutamylamino) butanoate, mevalonic acid], essential fatty acids (dihomo-α-linolenic acid, dihomolinoleic acid, 10-nitrolinoleic acid), and nucleosides (1-methylpseudouridine, 1-methyladenosine, 5-acetylamino-6-amino-3-methyluracil).

**TABLE 2:** Potential “biomarker” metabolites in the gut of rats subjected to middle cerebral artery occlusion and then treated with Hua-Feng-Dan.

No.	Metabolite	Adduct	Formula	VIP	Fold change	P-value	RT (sec)	m/z (Da)	MCAO relative to sham	HFD relative to MCAO
1	Isoleucyl-glutamate	ESI-	C11H20N2O5	3.7395	2.5023	0.007467	3.860466667	297.0860719	↓**	↑**
2	l-histidine	ESI-	C6H9N3O2	3.3481	1.5501	0.0000266	2.910783333	154.0613357	↓***	↑***
3	*N*-lactoyl-tyrosine	ESI-	C12H15NO5	3.1928	1.491	0.0005785	3.486933333	234.0774599	↓*	↑***
4	Prolyl-glutamate	ESI-	C10H16N2O5	2.8985	1.288	0.0000159	2.1204	289.1043458	↓***	↑***
5	*N*2-succinyl-l-ornithine	ESI+	C9H16N2O5	2.7493	1.3549	0.00000402	2.6917	197.0922616	↓***	↑***
6	*N*-palmitoyl cysteine	ESI-	C14H22O4	2.2736	1.1917	0.0001883	4.12965	299.1502338	↓***	↑***
7	*N*-eicosapentaenoyl tyrosine	ESI+	C29H39NO4	2.2714	1.2538	0.04553	5.259233333	466.2917694	↓*	↑*
8	*N*-myristoyl serine	ESI+	C17H33NO4	1.8209	1.1915	0.04869	3.001566667	354.2026037	↓*	↑*
9	Neuraminic acid	ESI+	C9H17NO8	1.652	1.1693	0.03706	1.885183333	250.0936791	↓**	↑*
10	Tryptophyl-valine	ESI-	C16H21N3O3	1.5117	1.1517	0.03807	3.252433333	340.1041964	↓***	↑*
11	*N*-arachidonoyl phenylalanine	ESI+	C29H41NO3	1.4403	1.0772	0.03423	5.647883333	452.3124366	↓***	↑*
12	*N*-hydroxy-l-tyrosine	ESI-	C9H11NO4	1.4192	1.1259	0.0461	2.086433333	196.0610941	↓**	↑*
13	Formiminoglutamic acid	ESI+	C6H10N2O4	1.2258	1.0766	0.03031	0.659583333	157.0609269	↓***	↑*
14	Indolylacryloylglycine	ESI+	C13H12N2O3	2.6872	1.3423	0.0000229	3.6687	227.0815858	↓***	↑***
15	*S*-(*N,N*-diethylcarbamoyl)glutathione	ESI+	C15H26N4O7S	2.0055	1.211	0.01691	3.6687	439.1866745	↓**	↑*
16	[4-((1Z)-2-(acetylamino)-3-{[1-(1,1′-biphenyl-4-ylmethyl)-2-oxoazepan-3-yl]amino}-3-oxoprop-1-enyl)-2-formylphenyl]acetic acid	ESI+	C33H33N3O6	3.5711	1.479	0.00000299	6.152633333	609.2711151	↓**	↑***
17	Phenoxyacetic acid	ESI-	C8H8O3	2.3235	1.2288	0.0004442	2.847966667	197.0450845	↓***	↑***
18	4-(glutamylamino) butanoate	ESI+	C9H16N2O5	1.8677	1.1971	0.02237	2.499783333	197.092293	↓**	↑*
19	Betalamic acid	ESI-	C9H9NO5	1.8272	1.1812	0.01588	3.185516667	210.0403707	↓**	↑*
20	Mevalonic acid	ESI-	C6H12O4	1.5096	1.0962	0.02435	2.19335	147.0653597	↓***	↑*
21	3-pyridylacetic acid	ESI-	C7H7NO2	1.5072	1.1504	0.04586	4.44565	273.0884249	↓***	↑*
22	2-hydroxy-3-methylbutyric acid	ESI-	C5H10O3	1.3419	1.0758	0.02808	2.3708	117.0545952	↓***	↑*
23	l-2-amino-3-(1-pyrazolyl) propanoic acid	ESI-	C6H9N3O2	1.2954	1.1164	0.02922	3.85495	154.0613238	↓***	↑*
24	Tetrahydrodipicolinate	ESI-	C7H9NO4	1.9509	1.228	0.03443	1.253466667	216.0510885	↓**	↑*
25	Dihomo-α-linolenic acid	ESI+	C20H34O2	3.1961	1.3878	0.00000456	5.733383333	348.2898665	↓***	↑***
26	1-methylpseudouridine	ESI-	C10H14N2O6	2.0696	1.2884	0.04183	2.12755	239.0672827	↓*	↑*
27	Dihomolinoleic acid	ESI+	C18H32O2	1.9652	1.2004	0.02263	6.201866667	313.2737588	↓***	↑*
28	10-nitrolinoleic acid	ESI+	C18H31NO4	1.3489	1.1215	0.04227	5.5701	308.2222508	↓***	↑*
29	Dihydroartemisinin	ESI-	C15H24O5	1.4443	1.1134	0.03475	4.6135	283.1552465	↓***	↑*
30	3-methylindole	ESI+	C9H9N	1.6699	1.2734	0.03927	4.678016667	132.0809435	↓***	↑*
31	Chenodeoxycholylthreonine	ESI+	C28H47NO6	1.4572	0.9174	0.02141	5.282533333	535.3747409	↑***	↓*
32	Phenylalanyl-prolyl-arginine nitrile	ESI+	C20H29N7O2	3.5046	0.5586	0.002597	5.282533333	422.2286168	↑***	↓**
33	Propylenediamine tetra-acetic acid	ESI+	C11H18N2O8	1.3507	0.9203	0.04406	5.62455	307.1112379	↑	↓*
34	l-2-Amino-3-oxobutanoic acid	ESI-	C4H7NO3	1.3245	0.9213	0.04815	3.5119	293.100749	↑	↓*
35	2′-deoxyadenosine 5′-phosphate	ESI-	C10H14N5O6P	2.7577	0.6502	0.04213	1.4989	330.0611481	↑**	↓*
36	2′-*O*-methyladenosine	ESI+	C11H15N5O4	2.7444	0.6529	0.00693	2.63745	264.1092031	↑	↓**
37	1-methyladenosine	ESI-	C11H15N5O4	2.6961	0.7185	0.009127	3.232133333	262.0947218	↑*	↓**
38	Deoxycytidylic acid	ESI-	C9H14N3O7P	2.225	0.7721	0.03284	1.03035	306.0498762	↑**	↓*
39	5-acetylamino-6-amino-3-methyluracil	ESI+	C7H10N4O3	1.4434	0.9286	0.00056	1.78335	163.0615097	↑*	↓***

ESI, electrospray ionization; RT, retention time; VIP, variable importance in the projection. *P < 0.05, **P < 0.01, ***P < 0.001.

**FIGURE 6:**
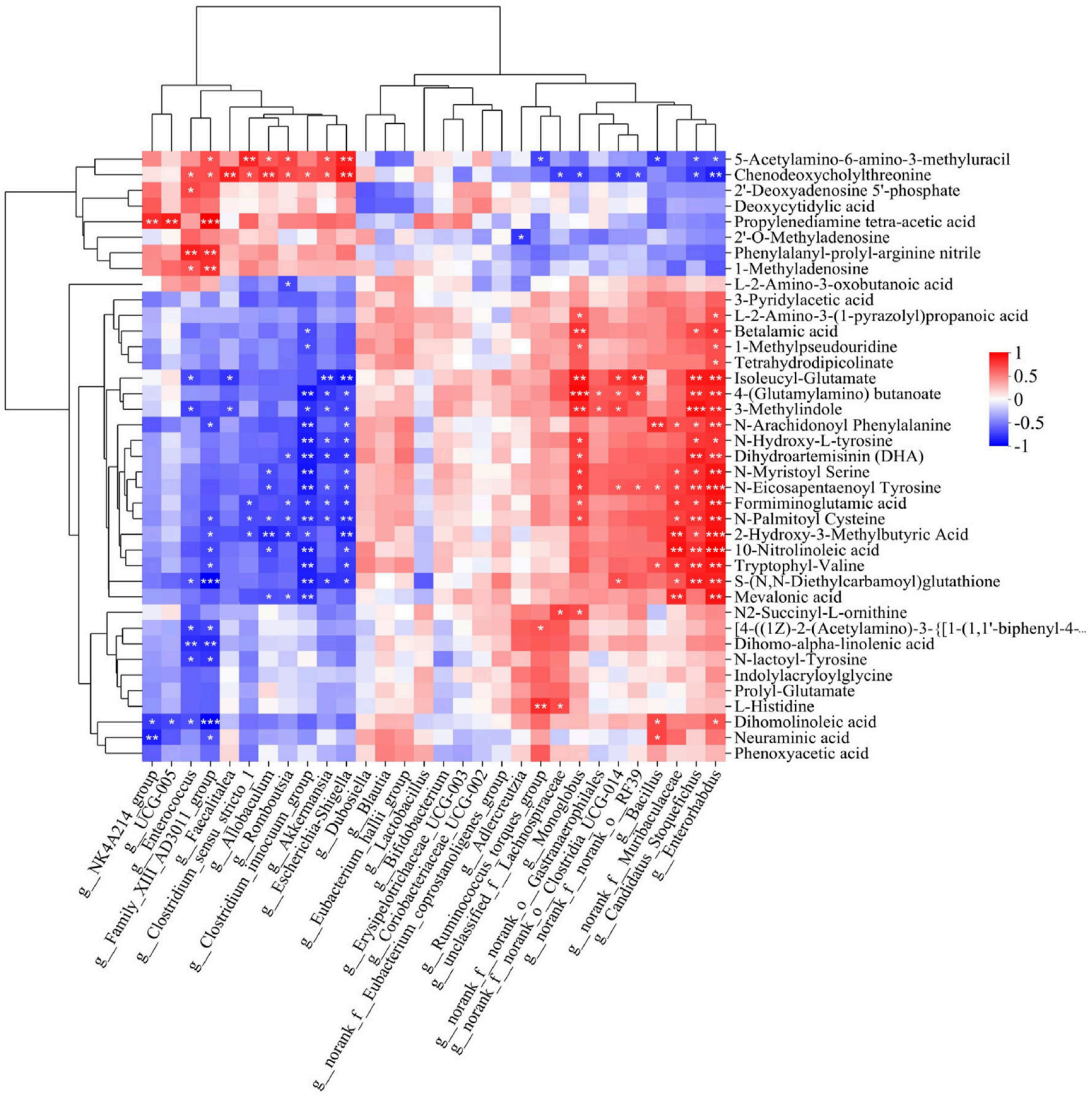
Heatmap of Spearman associations between relative abundance of microbial taxa and levels of differential metabolites in the gut. Correlation analysis between 39 differential metabolites whose levels in the gut were significantly altered by brain ischemia and were partially renormalized by Feng-Hua-Dan (see Table 2) with the top 30 gut bacteria in total abundance at the genus level. Cells are colored according to the Spearman correlation coefficient; red indicates a positive correlation, while blue indicates a negative correlation. Data are mean ± SD. *P < 0.05, **P < 0.01, ***P < 0.001.

We searched for Spearman correlations between the relative abundance of the 30 most abundant microbial taxa and the levels of the 39 potential metabolite biomarkers (Figure 6). Among taxa predominating after ischemic injury, the relative abundance of *g_Escherichia-Shigella*, *g__Clostridium_innocuum_group*, *g__Romboutsia*, *g__Allobaculum* and *g__Enterococcus* correlated positively with levels of 5-acetylamino-6-amino-3-methyluracil, chenodeoxycholylthreonine, 2′-deoxyadenosine 5′-phosphate, deoxycytidylic acid, phenylalanyl-prolyl-arginine nitrile, 1-methyladenosine, and 3-methylindole. Conversely, the abundance of these taxa correlated negatively with 4-(glutamylamino) butanoate, 2-hydroxy-3-methylbutyric acid, dihomo-α-linolenic acid, dihomolinoleic acid, and 10-nitrolinoleic acid. Among taxa predominating after treatment with Hua-Feng-Dan, the relative abundance of *g__unclassified_f__Lachnospiraceae*, *g__norank_f__noranko__Clostridia_UCG-014* and *g__Ruminococcus_torques_group* showed almost the opposite trend compared with the predominant bacteria in MCAO group. The abundance of these taxa correlated positively with 4-(glutamylamino) butanoate, 2-hydroxy-3-methylbutyric acid, and 10-nitrolinoleic acid.

### 3.4 Hua-Feng-Dan restores the integrity of the intestinal barrier after ischemic stroke in rats

**FIGURE 7:**
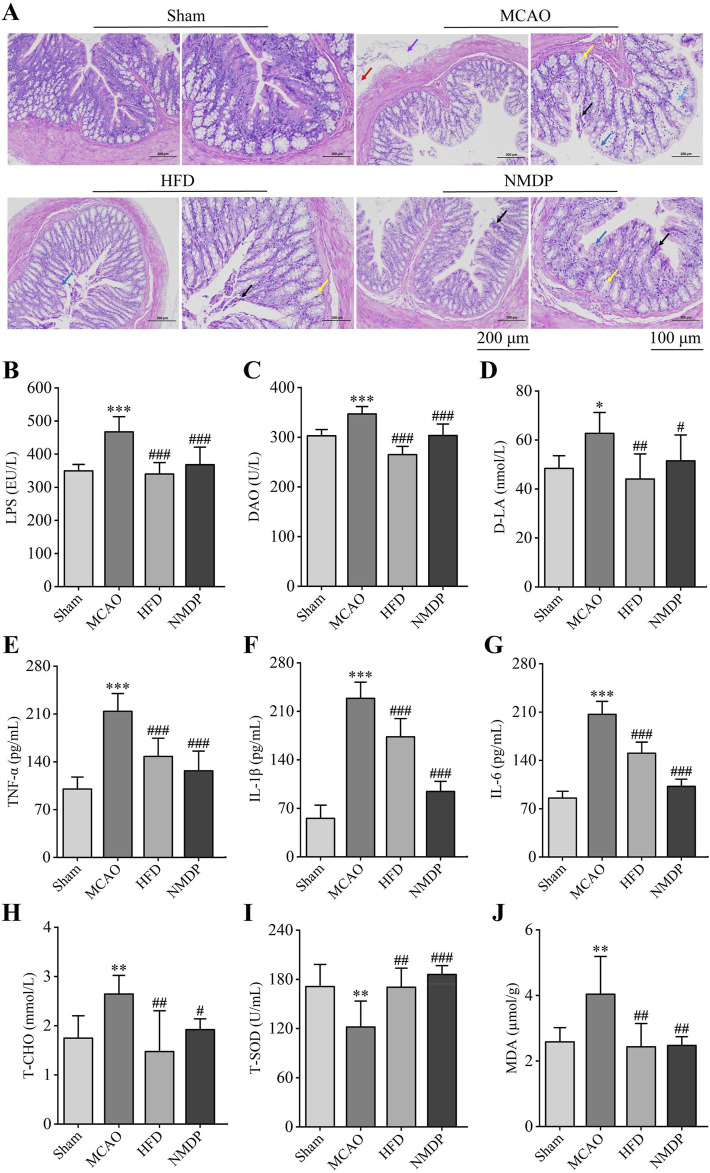
Hua-Feng-Dan restores the integrity of the intestinal barrier after ischemic stroke in rats. Animals were subjected to middle cerebral artery occlusion or sham surgery, then left untreated (MCAO, Sham) or treated with a high dose of Hua-Feng-Dan (HFD, 0.648 g/kg) and nimodipine (NMDP). **(A)** Representative photographs of colon H&E staining (n = 3). Red arrows indicate the presence of lymphocytic infiltration; blue arrows, edema or edematous mucosal epithelial cells; black arrows, necrosis of mucosal epithelial cells; yellow arrows, dilated intestinal glands; and purple arrows, vasodilation. Scale bar = 200 and 100 μm. **(B–D)** Comparison of levels of three indices of intestinal barrier permeability: lipopolysaccharide (LPS), diamine oxidase (DAO) and d-lactate (d-LA). **(E–G)** Comparison of levels of three pro-inflammatory factors: tumor necrosis factor (TNF)-α, interleukin (IL)-1β, and IL-6. **(H)** Comparison of levels of total cholesterol (T-CHO) as an index of dyslipidemia. **(I, J)** Comparison of levels of oxidative stress factors total superoxide dismutase (T-SOD) and malondialdehyde (MDA). Data are mean ± SD, n = 6 (excluding H&E staining). *P < 0.05, **P < 0.01, ***P < 0.001 vs. Sham group; ^#^ *P* < 0.05, ^##^ *P* < 0.01, ^###^ *P* < 0.001 vs. MCAO group (based on one-way ANOVA and Tukey’s multiple-comparisons *post hoc* test).

In the Sham group, the intestinal glands were tightly arranged, intact structure of the connective tissue, abundant goblet cells, and there was no obvious inflammatory cell infiltration. Hua-Feng-Dan treatment significantly improved ischemia/reperfusion-induced mucosal epithelial cell edema and necrosis, goblet cell reduction, intestinal gland dilatation and disorganization, lamina propria edema, plasma layer vasodilatation, and lymphocyte infiltration, while nimodipine did not alleviate the mucosal epithelial lesions (Figure 7A). The findings indicated that Hua-Feng-Dan had a protective effect on the integrity of the intestinal barrier.

MCAO significantly increased levels of lipopolysaccharide (LPS), diamine oxidase (DAO) and d-lactate (d-LA) in serum, consistent with translocation of gut microbiota into the circulation as a result of partial permeabilization of the gut epithelium (Figures 7B–D). Consistently, it significantly increased levels of the pro-inflammatory factors tumor necrosis factor (TNF)-α, interleukin (IL)-1β and IL-6 (Figures 7E–G); it increased total cholesterol (T-CHO) level, indicating dyslipidemia; and it increased the level of malondialdehyde (MDA) but decreased total level of superoxide dismutase (T-SOD), indicating greater oxidative stress (Figures 7H–J). All these ischemia-induced changes were significantly reversed by Hua-Feng-Dan or nimodipine, suggesting that the traditional Chinese medicine can repair ischemia-induced permeabilization of the intestinal barrier, which in turn mitigates systemic inflammatory responses and oxidative stress.

**FIGURE 8:**
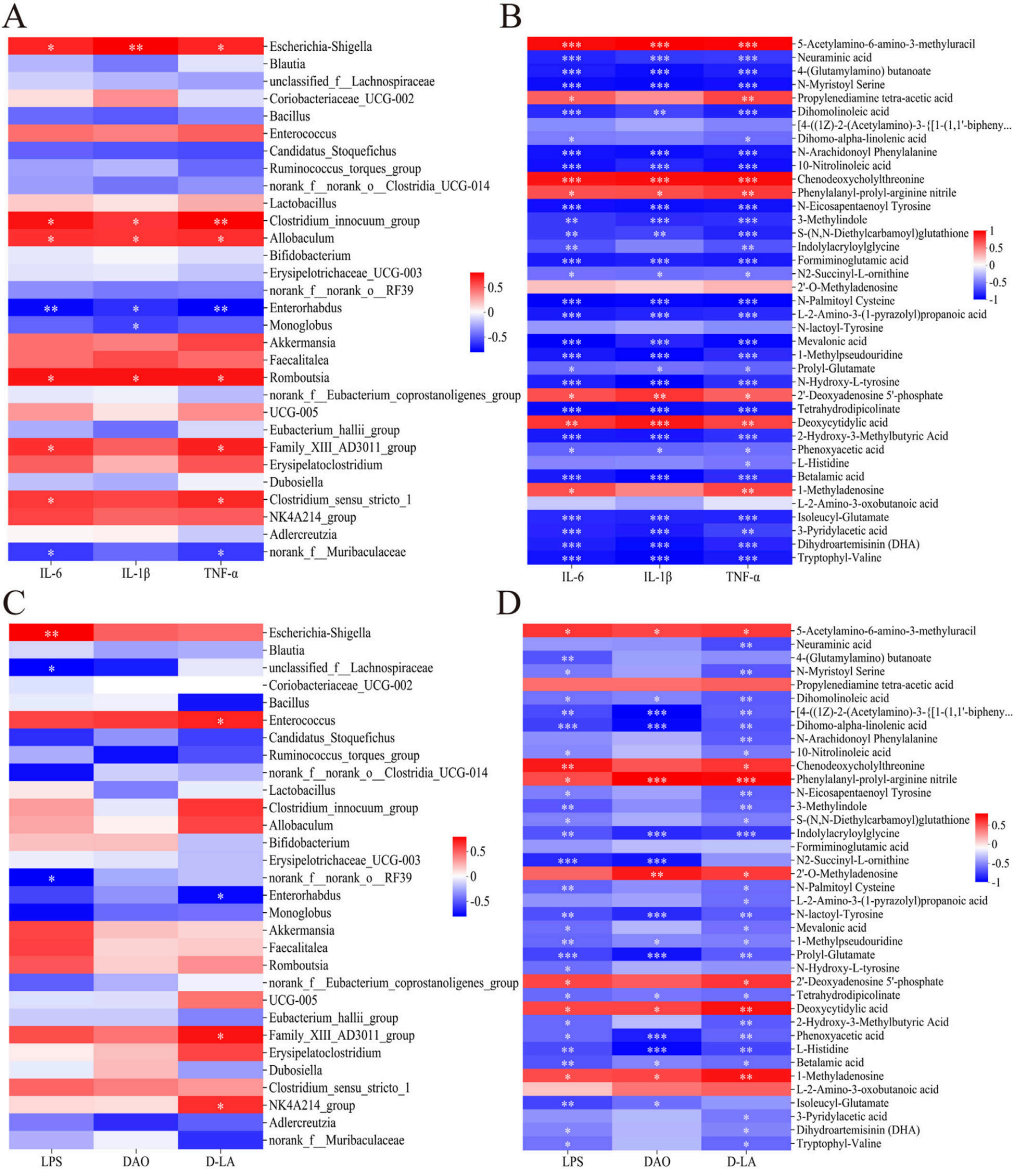
Heatmaps of Spearman associations **(A)** between relative abundance of microbial taxa in the gut and levels of pro-inflammatory cytokines in serum, **(B)** between levels of 39 differential metabolites of gut flora (see Table 2) and levels of pro-inflammatory cytokines in serum, **(C)** between relative abundance of microbial taxa in the gut and indices of intestinal barrier permeability in serum, and **(D)** between the 39 metabolites of gut flora in the gut and indices of intestinal barrier permeability in serum. Data are mean ± SD. *P < 0.05, **P < 0.01, ***P < 0.001.

We searched for Spearman correlations between the relative abundance of the 30 most abundant microbial taxa and the levels of the 39 potential metabolite biomarkers with the levels of the three markers of gut barrier permeability and three pro-inflammatory cytokines. Among taxa predominating after ischemic injury, the relative abundance of *Escherichia-Shigella*, *Clostridium_innocuum_group*, *Allobaculum*, *Romboutsia* and *Enterococcus* correlated positively with levels of the six marker molecules (Figures 8A, C). Conversely, among taxa predominating after treatment with Hua-Feng-Dan, the relative abundance of *unclassified_f__Lachnospiraceae* and *norank_f__norank_o__Clostridia_UCG-014* correlated negatively with the six marker levels.

Levels of the six marker molecules correlated negatively with levels of the metabolites 1-methylpseudouridine, 3-methylindole as well as metabolites from pathways involving metabolism of amino acids (e.g., prolyl-glutamate, *N*2-succinyl-l-ornithine, *N*-arachidonoyl phenylalanine), SCFAs [e.g., 3-pyridylacetic acid, l-2-amino-3-(1-pyrazolyl)propanoic acid, 2-hydroxy-3-methylbutyric acid, 4-(glutamylamino) butanoate, mevalonic acid], and essential fatty acids (e.g., dihomo-α-linolenic acid, dihomolinoleic acid, 10-nitrolinoleic acid) (Figures 8B, D). Conversely, marker levels correlated positively with levels of 5-acetylamino-6-amino-3-methyluracil, chenodeoxycholylthreonine, phenylalanyl-prolyl-arginine nitrile, 2′-deoxyadenosine 5′-phosphate, deoxycytidylic acid, propylenediamine tetra-acetic acid, and 1-methyladenosine. The analyses in Section 3.3 (Table 2) have shown that Hua-Feng-Dan downregulates these seven metabolites, while upregulating the related amino acids and SCFAs metabolites.

### 3.5 Hua-Feng-Dan helps brain metabolism return to normal following ischemic stroke in rats

**FIGURE 9:**
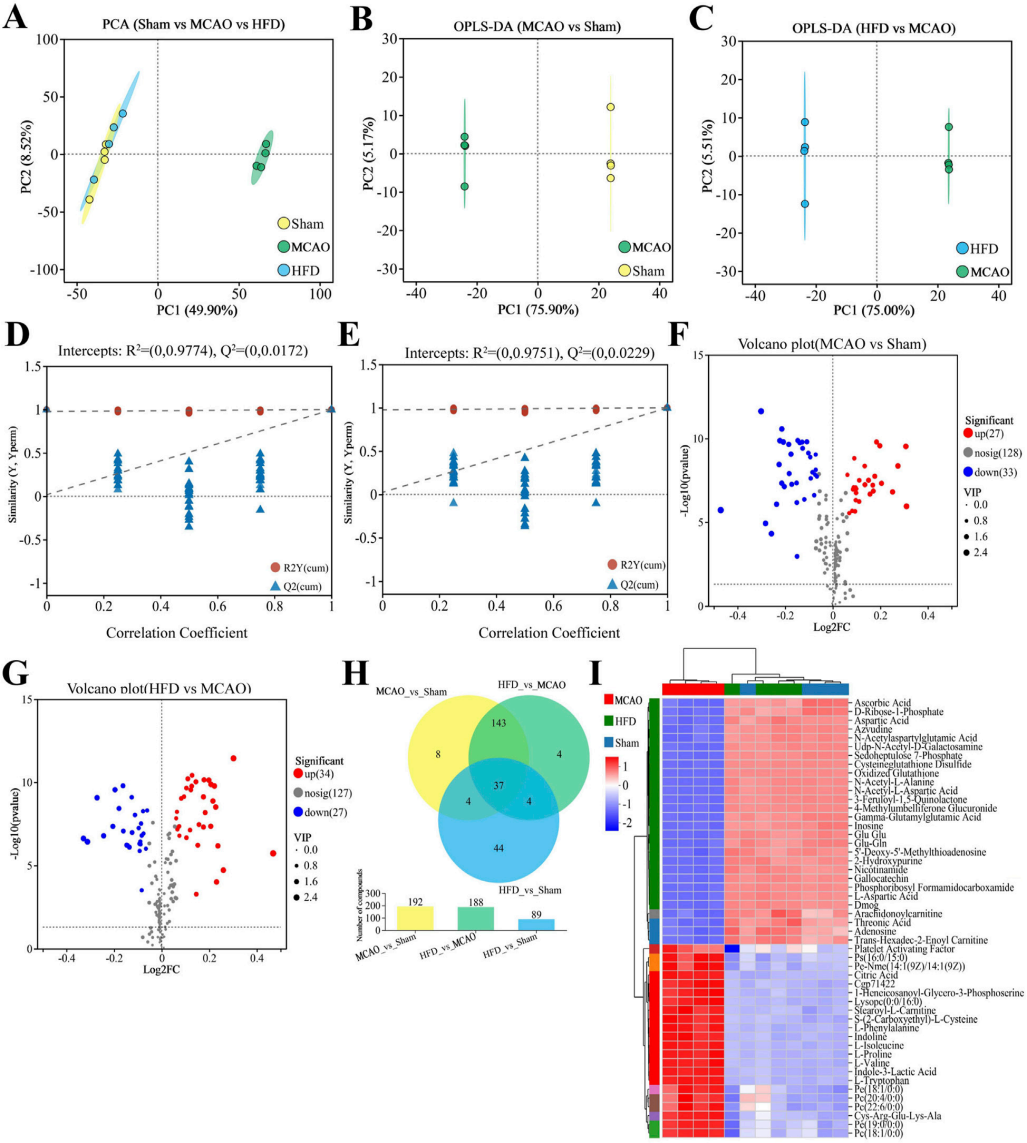
Hua-Feng-Dan renormalizes brain metabolism after ischemic stroke in rats. Animals were subjected to middle cerebral artery occlusion or sham surgery, then left untreated (MCAO, Sham) or treated with a high dose of Hua-Feng-Dan (HFD, 0.648 g/kg). Levels of metabolites were compared between Sham and MCAO animals, as well as between MCAO and HFD animals. **(A)** Principal component analysis (PCA) in negative ionization mode. **(B, C)** Orthogonal partial least-squares-discriminant analysis (OPLS-DA) in negative ionization mode. **(D, E)** OPLS-DA permutation testing with 200 permutations in negative ionization mode. *R* ^2^ measures goodness of fit, while Q^2^ measures predictive power of the model. **(F, G)** Volcano plots showing brain metabolites whose levels differed significantly in each pairwise comparison (negative ionization mode). **(H)** Veen plot analysis of differential metabolites in MCAO vs. Sham, HFD vs. MCAO, and HFD vs. Sham. **(I)** Metabolite cluster analysis of MCAO, HFD and Sham groups. Data are mean ± SD.

Metabolomics analysis of brain tissue was also carried out in several groups to investigate the alterations in rat brain metabolism. The MCAO group and the other two groups were clearly separated, but the HFD group and Sham were hardly separated at all (Figure 9A). Significant variations were seen in the metabolomic profiles of the sham-operated vs. untreated and treated vs. untreated animals (Figures 9B, C). Hua-Feng-Dan largely undid changes in brain metabolism brought on by ischemia. Permutation testing (Figures 9D, E) supports reliability of the finding by indicating that they are unlikely to be the result of overfitting. In fact, metabolite levels in the three groups varied significantly, as shown by volcano plots (Figures 9F, G). Metabolic data obtained in positive ion mode are presented in Supplementary Figure S5. There were 89 (44 unique) differential metabolites in HFD vs. Sham, 192 differential metabolites (8 unique) in MCAO vs. Sham, and 188 differential metabolites (4 unique) in HFD vs. MCAO (Figure 9H).

We identified a total of 38 potential biomarkers within brain of rats subjected to MCAO and treated with Hua-Feng-Dan (Table 3). These potential biomarkers encompass a variety of amino acids (e.g., citrulline, cys-arg-glu-lys-ala, glu glu, *N*-acetylaspartylglutamic acid, *N*-acetyl- l-alanine, l-tryptophan, l-phenylalanine, l-histidine, l-valine), SCFAs (2-hydroxy-3-methylbutyric acid, 2-aminohexanedioic acid), nucleosides (guanosine, isoguanosine, didanosine, adenine, 1-methyladenosine, dihydrothymine), glu-gln, and indole-3-carboxaldehyde. To explore the expression of brain metabolites within different groups, we performed metabolite clustering analysis. The expression of the two clusters was nearly opposite; the MCAO group gathered for the first cluster, whereas the HFD and Sham groups gathered for the second cluster (Figure 9I). In particular, MCAO was positively connected with l-phenylalanine, l-isoleucine, l-proline, l-valine, and l-tryptophan, whereas HFD was positively connected with l-aspartic acid, gamma-glutamylglutamic acid, *N*-acetyl-alanine, and inosine. These analyses demonstrated that the metabolism of brain underwent notable changes following IS, which were subsequently ameliorated through Hua-Feng-Dan intervention.

**TABLE 3:** Potential “biomarker” metabolites in the brain of rats subjected to middle cerebral artery occlusion and then treated with Hua-Feng-Dan.

No.	Metabolite	Adduct	Formula	VIP	Fold change	P-value	RT (sec)	m/z (Da)	MCAO relative to sham	HFD relative to MCAO
1	3-methylhistidine	ESI+	C7H11N3O2	2.0883	0.8484	0.00000001826	0.495316667	170.0918372	↑***	↓***
2	Citrulline	ESI+	C6H13N3O3	1.8579	0.8772	0.0000001405	0.503116667	176.1024226	↑***	↓***
3	(R)C(*S*)S-alliin	ESI+	C6H11NO3S	1.8285	0.8778	0.0000000149	5.465766667	160.042203	↑***	↓***
4	Cys-arg-glu-lys-ala	ESI+	C23H43N9O8S	1.7376	0.9095	0.0001518	7.19095	570.2805279	↑***	↓***
5	Glu glu	ESI+	C10H16N2O7	1.718	1.0913	0.0000000005884	0.556966667	277.1020443	↓***	↑***
6	l-aspartic acid	ESI+	C4H7NO4	1.5298	1.0682	0.0000000002642	0.870733333	134.0444298	↓***	↑***
7	l-isoleucine	ESI+	C6H13NO2	1.28	0.9563	0.000000008045	1.4378	132.1015102	↑***	↓***
8	*N*-acetyl- l-alanine	ESI-	C5H9NO3	1.3122	1.0747	0.0000000002206	0.8702	130.050322	↓***	↑***
9	l *-*valine	ESI+	C5H11NO2	1.2529	0.9562	0.00000005576	0.5726	118.0861386	↑***	↓***
10	l-tryptophan	ESI+	C11H12N2O2	1.2189	0.9562	0.00000004689	2.227116667	205.0964816	↑***	↓***
11	l-phenylalanine	ESI+	C9H11NO2	1.139	0.9644	0.0000002233	1.808683333	166.0858009	↑***	↓***
12	l-histidine	ESI+	C6H9N3O2	1.0787	0.9621	0.000001623	0.487683333	156.0763379	↑***	↓***
13	Pro ile	ESI+	C11H20N2O3	1.1126	1.0551	0.000019250	2.053416667	229.1537792	↓***	↑***
14	l-proline	ESI+	C5H9NO2	1.2295	0.9568	0.000000111	0.541383333	116.0705048	↑***	↓***
15	l-tyrosine	ESI+	C9H11NO3	1.0777	0.9665	0.0000001159	1.3588	182.0806663	↑***	↓***
16	*N*-acetylaspartylglutamic acid	ESI-	C11H16N2O8	1.0738	1.0469	0.00000006684	1.531383333	303.083942	↓***	↑***
17	Mycobactins	ESI+	C27H37N5O10	1.5423	0.9131	0.0005661	7.183116667	614.2442274	↑***	↓***
18	1-methyladenosine	ESI+	C11H15N5O4	1.1285	0.9567	0.0000009784	1.066516667	282.1185588	↑***	↓***
19	5′-deoxy-5′-methylthioadenosine	ESI+	C11H15N5O3S	1.279	1.0511	0.0000001532	2.1246	298.0958038	↓***	↑***
20	Adenosine	ESI+	C10H13N5O4	1.5164	1.0752	0.00000006888	1.56385	268.1030718	↓***	↑***
21	Isoguanosine	ESI+	C10H13N5O5	1.5468	1.0974	0.0000003552	1.595816667	284.0978085	↓***	↑***
22	2′-deoxycytidine	ESI+	C9H13N3O4	1.1495	0.9442	0.0002399	0.964816667	228.096957	↑***	↓***
23	Didanosine	ESI+	C10H12N4O3	1.1072	0.9515	0.000006115	1.548166667	278.1237423	↑***	↓***
24	5′-guanylic acid	ESI-	C10H14N5O8P	1.7417	1.1779	0.0000006661	1.006233333	362.0514248	↓***	↑***
25	Guanosine	ESI-	C10H13N5O5	1.2331	1.086	0.00000007437	1.6778	282.0849323	↓***	↑***
26	2-hydroxypurine	ESI+	C5H4N4O	1.3279	1.0474	0.000000008121	1.595816667	137.045413	↓***	↑***
27	Adenine	ESI+	C5H5N5	1.1493	1.0461	0.00000002554	0.807766667	136.0614006	↓***	↑***
28	Dihydrothymine	ESI+	C5H8N2O2	1.1804	1.0501	0.00000232	0.518533333	129.0656662	↓***	↑***
29	2-aminohexanedioic acid	ESI+	C6H11NO4	1.1663	0.9529	0.0000008408	0.556966667	162.07557	↑***	↓***
30	2-hydroxy-3-methylbutyric acid	ESI-	C5H10O3	1.0901	0.9395	0.0000003643	2.775633333	117.0550123	↑***	↓***
31	Histamine	ESI+	C5H9N3	1.8023	0.8463	0.00002835	0.416883333	112.0868687	↑***	↓***
32	Aceglutamide	ESI+	C7H12N2O4	1.5508	0.9148	0.0000001164	0.839066667	189.0863141	↑***	↓***
33	*S*-lactoylglutathione	ESI-	C13H21N3O8S	1.0733	0.9483	0.0000001199	1.6778	378.0982509	↑***	↓***
34	Cyclophosphamide	ESI+	C7H15Cl2N2O2P	1.2953	0.9376	0.00000004788	0.38555	261.029684	↑***	↓***
35	Indole-3-carboxaldehyde	ESI+	C9H7NO	1.061	0.9581	0.000002568	2.227116667	146.0596071	↑***	↓***
36	Arachidonoylcarnitine	ESI+	C27H45NO4	1.1075	1.0382	0.000001235	5.669866667	448.3403549	↓***	↑***
37	Glu-gln	ESI+	C10H17N3O6	2.1446	1.1515	0.00000000382	0.549133333	276.1179992	↓***	↑***
38	Oxidized glutathione	ESI-	C20H32N6O12S2	2.234	1.2327	0.000000000003593	1.573166667	611.1446294	↓***	↑***

ESI, electrospray ionization; RT, retention time; VIP, variable importance in the projection. ***P < 0.001.

## 4 Discussion

Our studies in a rat model of IS suggest that the therapeutic effects of the traditional Chinese medicine Hua-Feng-Dan are due at least partly to renormalization of ischemia-induced dysbiosis of gut flora and concomitant renormalization of gut microbial metabolism. As a result, reduction in intestinal permeability, fewer gut bacteria invade the bloodstream, mitigating systemic inflammatory responses, dyslipidemia, oxidative stress, and metabolic disturbances in brain.

Our research confirms and extends previous work linking dysbiosis of gut flora to severity of ischemic injury (Xian et al., 2022). In our rats, MCAO was associated with high relative abundance of detrimental bacteria and opportunistic pathogens such as *Enterobacteriaceae*, *Erysipelotrichaceae* and *Enterococcaceae* at the family level. Higher abundance of *Enterobacteriaceae* has been linked to stronger systemic inflammation and higher risk of poor prognosis in stroke patients (Qian et al., 2023). Ischemic/reperfusion in our rats was also associated with high abundance of *Escherichia-Shigella* and *Enterococcus* at the genus level. The cell walls of *Escherichia-Shigella*, a Gram-negative bacterium in the *Proteobacteria* phylum, contains lipopolysaccharide (LPS), which permeabilizes the gut and blood-brain barrier, leading to inflammatory responses in the gut and central nervous system (Chen Y. et al., 2019), which are mediated in part by IL-1β (Mirsepasi-Lauridsen et al., 2019). *Enterococcus*, for its part, contributes to inflammatory cascades by releasing nitric oxide synthase and activating macrophages (Wu et al., 2013). Conversely, ischemic brain injury in our animals was associated with reduced abundance of bacteria that produced SCFAs such as *Lachnospiraceae*, *Butyricicoccaceae*, *Blautia*, *Akkermansia*, *Ruminococcus*, *Coprococcus* and *Prevotella,* as reported in IS patients (Li et al., 2019; Tan et al., 2021).

Our analyses suggest that Hua-Feng-Dan can renormalize the flora with therapeutic effects. For example, the traditional Chinese medicine can reverse the ischemia-induced decrease in abundance of *Firmicutes* bacteria such as *Lachnospiraceae,* which produces the short-chain fatty acid butyrate and higher abundance of which is associated with lower risk of stroke (Zeng et al., 2019). The abundance of this phylum, comprising mostly probiotic bacteria, is known to be reduced in the gut in rats after ischemic/reperfusion and in stroke patients with cognitive impairment (Ling et al., 2020; Xian et al., 2022). Increasing its abundance has been shown to improve intestinal barrier function and dampen inflammatory responses (Yu et al., 2021; Zhang et al., 2021). Hua-Feng-Dan decreased the abundance of pathogenic bacteria such as *Escherichia-Shigella* and *Enterococcus* in our animals, while increasing the abundance of beneficial bacteria such as *Candidatus_Stoquefichus*, *Ruminococcus_torques_group*, *unclassified__f__Lachnospiracea* and *Akkermansia*. *Akkermansia*, a member of *Verrucomicrobiota*, produces SCFAs, exerts anti-inflammatory properties (Zhang et al., 2017) and can promote intestinal barrier function (Hua et al., 2008; Li et al., 2021).

In these ways, our results suggest that augmenting populations of bacteria that produce SCFAs is a therapeutic approach against IS. SCFAs such as butyrate can maintain gut barrier integrity and mitigate colitis by inhibiting pro-inflammatory cytokines (Tan et al., 2014; Zhao et al., 2020). The PICRUSt algorithm linked Hua-Feng-Dan to upregulation of butanoate metabolism, leading to higher levels of 4-(glutamylamino) butanoate and 2-hydroxy-3-methylbutyric acid, which correlated in turn with lower levels of pro-inflammatory cytokines. Butyrate on its own or bacteria rich in SCFAs can improve nervous system function such as after IS (Bourassa et al., 2016; Chen R. et al., 2019), so we hypothesize that Hua-Feng-Dan may help mitigate IS injury by increasing the relative abundance of probiotic gut bacteria that produce SCFAs such as *Lachnospiraceae*, *unclassified__f__Lachnospiracea*, *Ruminococcus_torques_group* and *Akkermansia*. The therapeutic efficacy of boosting these fatty acids should be explored in individuals and animal models using targeted metabolomics to measure their levels.

In renormalizing the composition of gut flora after IS, Hua-Feng-Dan also appears to renormalize gut microbial and cerebral metabolism. In particular, the medicine renormalizes the metabolism of amino acids, which together with their metabolites are known to drive excessive inflammatory responses after acute cerebral ischemia (Leppkes and Neurath, 2020; Zheng et al., 2019). Numerous amino acids, including l-isoleucine, l-valine, l-phenylalanine, l-proline, and l-tyrosine, were shown to be elevated in the brain of MCAO rats, which were dramatically reduced following administration of Hua-Feng-Dan. In our rat model, Hua-Feng-Dan upregulated various amino acid metabolites of gut flora (like prolyl-glutamate, *N*2-succinyl-l-ornithine, *N*-arachidonoyl phenylalanine) whose increases were associated with decreases in levels of pro-inflammatory cytokines in serum. While the pro-inflammatory cytokines TNF-α and IL-6 upregulate arginase activity, affecting degradation of arginine and tryptophan metabolism (Baek et al., 2020; Chen et al., 2022), the PICRUSt algorithm predicted that Hua-Feng-Dan upregulates biosynthesis of arginine, and tryptophan metabolism is significantly enriched in KEGG topology analysis. The nonessential amino acid arginine can enhance cerebral blood flow and metabolism while reducing infarct volume in a rat model of cerebral ischemia (He et al., 1995; Temiz et al., 2003). Arginine supplementation has been shown to mitigate gut injury and strengthen immune responses by promoting gut health through gut microbiota (Wu et al., 2020; Xie et al., 2020), and it can protect rats against cerebral infarction induced by MCAO (Kondoh et al., 2010). Tryptophan metabolites, for their part, can reduce harmful behavior by gut flora (Li et al., 2014) and mitigate astrocyte activation to suppress neuroinflammatory responses in the central nervous system (Rothhammer et al., 2016). Cys-arg-glu-lys-ala and l-tryptophan were discovered to be considerably increased in the MCAO group in the metabolomic analysis of brain tissue. These findings may suggest that arginine and tryptophan are neuroprotective targets for Hua-Feng-Dan treatment of IS.

Our analyses suggest that Hua-Feng-Dan dampens inflammatory responses by upregulating dihomo-α-linolenic acid, dihomolinoleic acid, and 10-nitrolinoleic acid, and lower levels of these metabolites correlated with higher levels of pro-inflammatory cytokines in our rat model. Indeed, linoleic acid (LA) and α-linolenic acid (ALA), which are essential fatty acids obtained from food, have been shown to decrease the levels of TNF-α, IL-1β and IL-6 and thereby mitigate neuroinflammation (Boneva et al., 2011; Zhang et al., 2011). LA can replace fatty acids (Luc et al., 2003; Ristić-Medić et al., 2001) and provide energy to the brain (Panov et al., 2014). Higher intake of LA can decreased the risk of stroke (Al-Khudairy et al., 2015; Iacono et al., 1983) or mitigate stroke-induced injury (Tsai et al., 1994; Venø et al., 2017) and is associated with a reduced risk of mortality from neurodegenerative disorders (Kim and Song, 2024). Purine metabolism-related guanosine and inosine have been shown to have neuroprotective properties. Hua-Feng-Dan showed substantial positive correlation with inosine and markedly reversed levels of guanosine, isoguanosine, 5′-guanylic acid, and didanosine caused by MCAO in brain metabolism. Guanosine possesses anti-inflammatory and antioxidant properties in ischemic brain injury (Dal-Cim et al., 2013; Dal-Cim et al., 2011). It can also control glutaminergic excitatory toxicity-induced neuronal damage. Inosine improves behavioral outcomes after a stroke (Chen et al., 2002; Smith et al., 2007). Our analyses justify further research into the potential therapeutic efficacy of boosting levels of LA, ALA, and guanosine against IS.

Hua-Feng-Dan has been used to treat IS for about 380 years. There is some public concern about its safety due to the presence of cinnabar and realgar in the preparation of Hua-Feng-Dan. In the 2020 edition of the Chinese Pharmacopoeia, the recommended daily dosages for cinnabar and realgar are 0.100 ∼ 0.500 g and 0.050 ∼ 0.100 g, respectively. The daily dosages of cinnabar and realgar in Hua-Feng-Dan are about 0.046 ∼ 0.087 g and 0.051 ∼ 0.096 g respectively. None of the cinnabar and realgar exceeded the dosage prescribed by Chinese Pharmacopoeia. Clinical studies have shown that the Qing Huang Powder (daily dose of 0.1 g of realgar) is effective and safe for treating patients with myelodysplastic syndrome (Zhu et al., 2017). No significant toxicity was observed in the clinical dose range of Hua-Feng-Dan. However, excessive exposure to arsenic and mercury may still pose risks, especially with long-term or excessive medication. A recommended treatment course for Hua-Feng-Dan lasts 18 days and necessitates medical supervision. Therefore, strict adherence to the provided guidelines is strongly advised.

## 5 Conclusion

Our analyses suggest that Hua-Feng-Dan can mitigate brain damage, inflammatory responses, and brain metabolic disorders after IS by restoring gut barrier function and renormalizing the composition and metabolism of gut microbiota.

## Data Availability

The datasets presented in this study can be found in online repositories. The names of the repository/repositories and accession number(s) can be found below: MTBLS10818 (Metabolights) and PRJNA1129823 (SRA).
