# Supplementary material for: Therapeutic effects of traditional Chinese medicine Hua-Feng-Dan in a rat model of ischemic stroke involve renormalization of gut microbiota
Source: Front Pharmacol. 2025 Jan 27;16:1485340. doi: 10.3389/fphar.2025.1485340 (PMC11808003; doi:10.3389/fphar.2025.1485340)
Supplement: Supplementary file 2 [file DataSheet1.docx]

Supplementary Materials

# Supplementary Methods

## LC-MS analysis of Hua-Feng-Dan

Aliquots of Hua-Feng-Dan weighing 100 ± 5 mg were placed into 2-mL microcentrifuge tubes, to which were added a grinding bead with diameter of 6 mm and 400 µL of methanol/water (4/1, v/v) containing the following internal standard L-2-chlorophenylalanine (0.02 mg/mL, Adamas-beta). The sample was ground for 6 min using a freezing tissue grinder (-10 °C, 50 Hz), cryo-sonicated for 30 min at 5 °C (40 kHz), left at -20 °C for 30 min, then centrifuged for 15 min at 13,000 g at 4 °C.

The resulting supernatant was injected onto a Vanquish Horizon ultra-high performance liquid chromatography (Thermo, MA, USA) equipped with a ACQUITY UPLC BEH C18 column (100 mm long, 2.1 mm inner diameter, 1.7 µm pore size; Waters, Milford, MA, USA). The following chromatographic parameters were used: the mobile phase A was 2% acetonitrile in water (containing 0.1% formic acid), while mobile phase B was acetonitrile (containing 0.1% formic acid), injection volume, 3 μL; column temperature, 40 °C.

The gradients were as follows: 2%-2%B, 0-0.5 min; 2%-25%B; 0.5-3.5 min; 25%-35%B, 3.5-7.5 min; 35%-50% B, 7.5-11 min; 50%-95%B, 11-13 min; 95%-95% B, 13-14.4 min; 95%-2% B, 14.4-14.5 min; 2%-2% B, 14.5-16 min.

## The detailed parameters for 16S rRNA sequencing analysis

The total genomic DNA of the microbial community was extracted following the protocol of the Fast Pure Stool DNA Isolation Kit (MJYH, Shanghai, China). The integrity of the extracted genomic DNA was assessed using 1% agarose gel electrophoresis. DNA concentration and purity were determined using NanoDrop2000 (Thermo, USA).

PCR amplification of the V3-V4 variable region of the 16S rRNA gene was conducted using the upstream primer 338F (5'-ACTCCTACGGGGAGGCAGCAG-3') and downstream primer 806R (5'-GGACTACHVGGGGTWTCTAAT-3'), which carried Barcode sequences. The amplification procedure is as follows:

pre-denaturation at 95 ℃ for 3 min, followed by 27 cycles of denaturation at 95 ℃ for 30 s, annealing at 55 ℃ for 30 s, and extension at 72 ℃ for 45 s. This was then followed by a stable extension at 72 ℃ for an additional 10 min before finally being stored at 10 ℃ (PCR instrument: ABI GeneAmp® 9700 model). The PCR reaction system consisted of the following components: 5×TransStart FastPfu Buffer 4 μL, 2.5 mM dNTPs 2 μL, Forward Primer (5 uM) 0.8 μL, Reverse Primer (5 uM) 0.8 μL, TransStart FastPfu DNA Polymerase 0.4 μL, BSA 0.2 μL, and10 ng of Template DNA, made up to 20 μL.

Three replicates were performed for each sample. PCR products from the same sample were combined and then recovered using a 2% agarose gel for PCR product retrieval and purification, 2% agarose gel electrophoresis for detecting band fragment sizes, and the Quantus™ Fluorometer (Promega, USA) for quantifying the recovered products.

## The detailed parameters for LC-MS analysis

The following gradient program was used for elution conditions in positive ion mode: 100% A 0-3 min, 80% -65% A 3-4.5 min, 65% -0% A 4.5-5 min, 0% -0% A 5 -6.3 min, 0% -100% A 6.3-6.4 min, 100% -100% A 6.4-8 min.

For elution conditions in negative ion mode, the following gradients were employed: 100% A 0-1.5 min, 95%-90% A 1.5-2 min, 90%-70% A 2-4.5 min, 70%-0% A 4.5 -5 min, 0% -0% A 5-6.3 min, 0% -100% A 6.3-6.4 min, 100% -100% A 6.4-8 min.

## LC-MS analysis of Hua-Feng-Dan


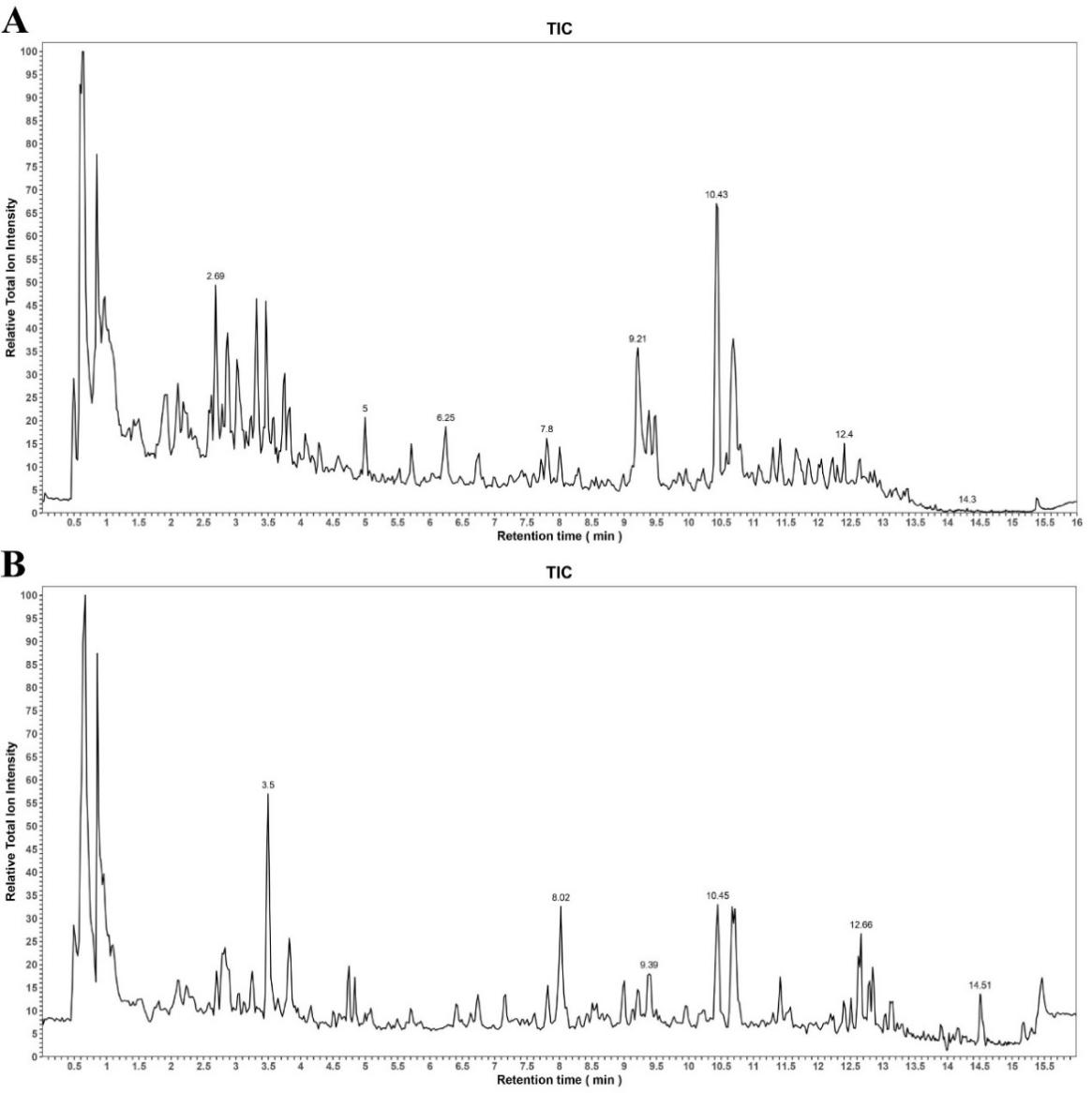


**Supplementary Figure S1** Total ion chromatogram of Hua-Feng-Dan water-soluble sample.

Total ion currents (TIC) chromatograms of the major constituents in the Hua-Feng-Dan water-soluble sample. These compounds are detected under negative (C) and positive (D) mode, respectively.

## Internal standard stability assessment for LC-MS analysis


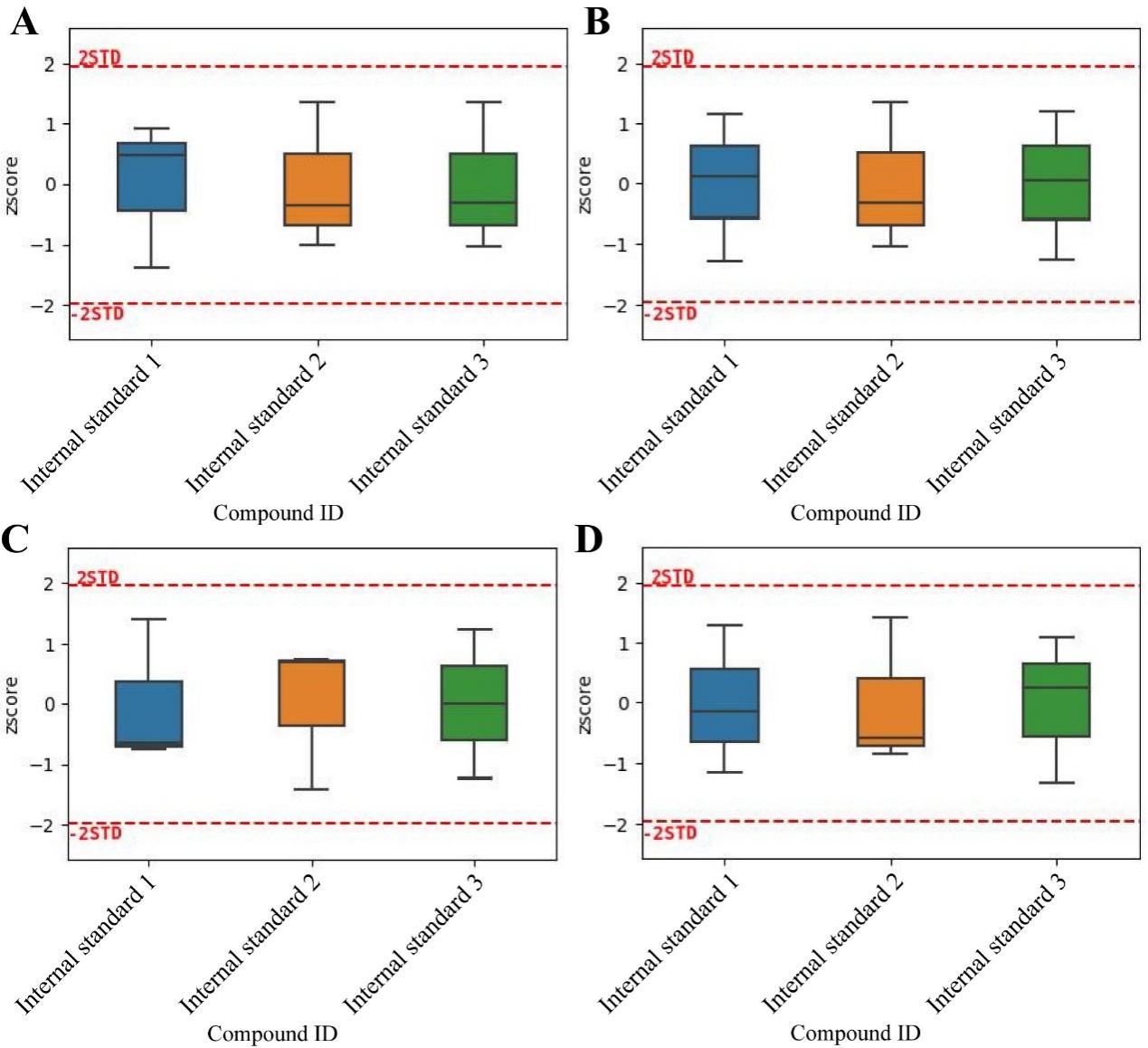


**Supplementary Figure S2** Internal standard stability assessment of cecum contents and brain tissues.

(A) Boxplot of z-score of internal standards in ESI (+) mode for QC samples of cecal contents. (B) Boxplot of z-score of internal standards in ESI (-) mode for QC samples of cecal contents. (C) Boxplot of z-score of internal standards in ESI (+) mode for QC samples of brain tissues. (D) Boxplot of z-score of internal standards in ESI (-) mode for QC samples of brain tissues.

## Total ion chromatogram for LC-MS analysis


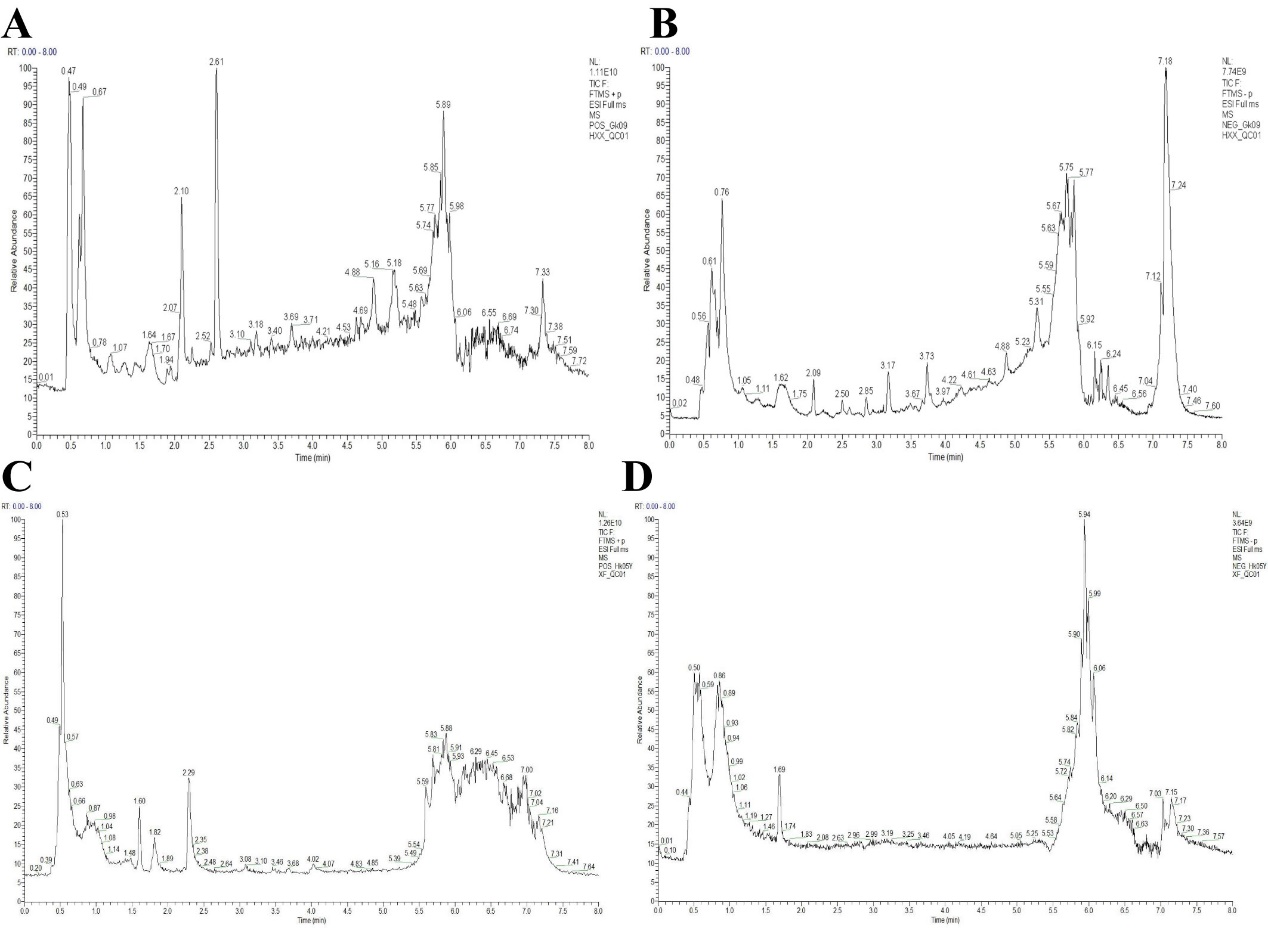


**Supplementary Figure S3** Total ion chromatogram of cecum contents and brain tissues.

(A) Total ion flow diagram of ESI (+) for the QC samples of cecal contents. (B) Total ion flow diagram of ESI (-) for the QC samples of cecal contents. (C) Total ion flow diagram of ESI (+) for the QC samples of brain tissues. (D) Total ion flow diagram of ESI (-) for the QC samples of brain tissues.

## LC-MS analysis of cecum contents in positive ionization mode


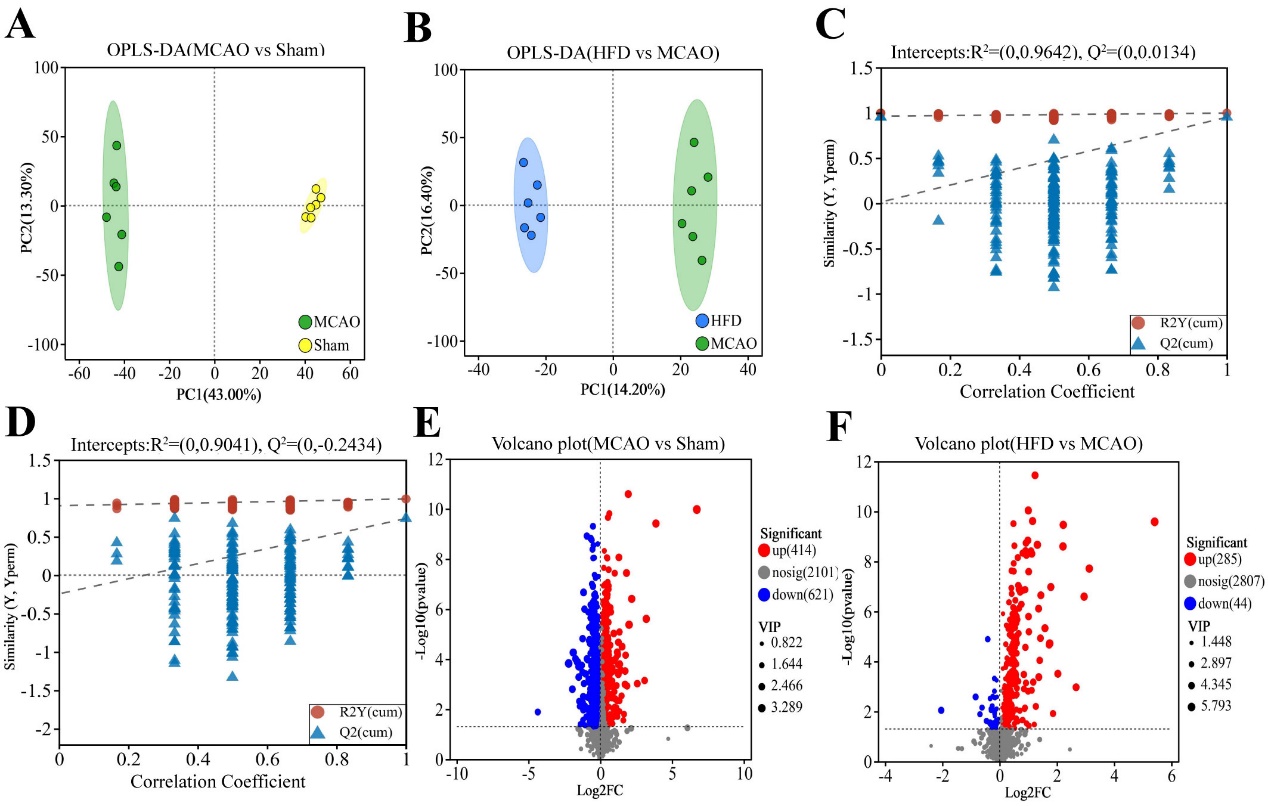


**Supplementary Figure S4** Hua-Feng-Dan renormalizes gut microbial metabolism after ischemic stroke in rats.

(A-B) Orthogonal partial least-squares-discriminant analysis (OPLS-DA) in positive ionization mode. (C-D) OPLS-DA permutation testing with 200 permutations in positive ionization mode. R2 measures goodness of fit, while Q2 measures predictive power of the model. (E-F) Volcano plots showing gut metabolites whose levels differed significantly in each pairwise comparison (positive ionization mode).

## LC-MS analysis of brain tissues in positive ionization mode


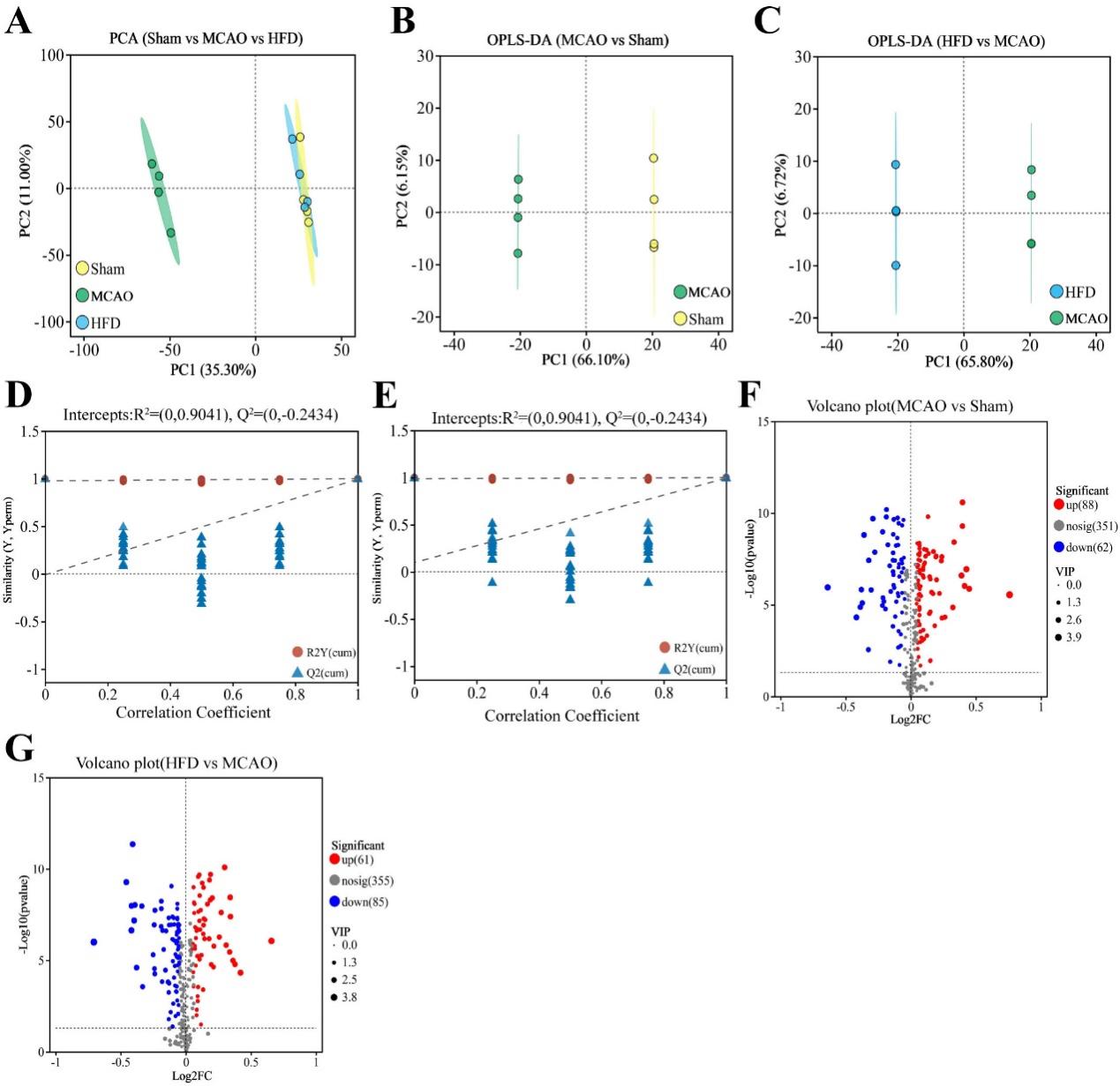


**Supplementary Figure S5** Hua-Feng-Dan renormalizes brain metabolism after ischemic stroke in rats.

(A) Principal component analysis (PCA) in positive ionization mode. (B-C) Orthogonal partial least-squares-discriminant analysis (OPLS-DA) in positive ionization mode. (D-E) OPLS-DA permutation testing with 200 permutations in positive ionization mode. R2 measures goodness of fit, while Q2 measures predictive power of the model. (F-G) Volcano plots showing brain metabolites whose levels differed significantly in each pairwise comparison (positive ionization mode).
